# A Randomised Controlled Trial to Reduce Sedentary Time in Young Adults at Risk of Type 2 Diabetes Mellitus: Project STAND (Sedentary Time ANd Diabetes)

**DOI:** 10.1371/journal.pone.0143398

**Published:** 2015-12-01

**Authors:** Stuart J. H. Biddle, Charlotte L. Edwardson, Emma G. Wilmot, Thomas Yates, Trish Gorely, Danielle H. Bodicoat, Nuzhat Ashra, Kamlesh Khunti, Myra A. Nimmo, Melanie J. Davies

**Affiliations:** 1 School of Sport, Exercise & Health Sciences, Loughborough University, Loughborough, United Kingdom; 2 Leicester Diabetes Research Centre, University of Leicester, Leicester, United Kingdom; 3 School of Health Sciences, University of Stirling, Stirling, United Kingdom; 4 The NIHR Leicester Loughborough Diet, Lifestyle and Physical Activity Biomedical Research Unit, Leicester and Loughborough, United Kingdom; Indiana University Richard M. Fairbanks School of Public Health, UNITED STATES

## Abstract

**Aims:**

Type 2 diabetes mellitus (T2DM), a serious and prevalent chronic disease, is traditionally associated with older age. However, due to the rising rates of obesity and sedentary lifestyles, it is increasingly being diagnosed in the younger population. Sedentary (sitting) behaviour has been shown to be associated with greater risk of cardio-metabolic health outcomes, including T2DM. Little is known about effective interventions to reduce sedentary behaviour in younger adults at risk of T2DM. We aimed to investigate, through a randomised controlled trial (RCT) design, whether a group-based structured education workshop focused on sitting reduction, with self-monitoring, reduced sitting time.

**Methods:**

Adults aged 18–40 years who were either overweight (with an additional risk factor for T2DM) or obese were recruited for the Sedentary Time ANd Diabetes (STAND) RCT. The intervention programme comprised of a 3-hour group-based structured education workshop, use of a self-monitoring tool, and follow-up motivational phone call. Data were collected at three time points: baseline, 3 and 12 months after baseline. The primary outcome measure was accelerometer-assessed sedentary behaviour after 12 months. Secondary outcomes included other objective (activPAL) and self-reported measures of sedentary behaviour and physical activity, and biochemical, anthropometric, and psycho-social variables.

**Results:**

187 individuals (69% female; mean age 33 years; mean BMI 35 kg/m^2^) were randomised to intervention and control groups. 12 month data, when analysed using intention-to-treat analysis (ITT) and per-protocol analyses, showed no significant difference in the primary outcome variable, nor in the majority of the secondary outcome measures.

**Conclusions:**

A structured education intervention designed to reduce sitting in young adults at risk of T2DM was not successful in changing behaviour at 12 months. Lack of change may be due to the brief nature of such an intervention and lack of focus on environmental change. Moreover, some participants reported a focus on physical activity rather than reductions in sitting per se. The habitual nature of sedentary behaviour means that behaviour change is challenging.

**Trial Registration:**

Controlled-Trials.com ISRCTN08434554

## Introduction

Type 2 diabetes (T2DM) is one of the most prevalent and costly chronic diseases globally. In the UK alone, 6% of the adult population are estimated to have the disease with a treatment cost that equates to around 10% of the National Health Service budget, and this relative cost is projected to increase to 17% by 2035 [1]. The increasing burden of T2DM is driven by our modern environments where sedentary lifestyles and ready access to energy dense foods now represent the norm. This modern environmental reality, particularly in younger populations, has precipitated a substantial shift in the demographic profile of T2DM. Once the disease only occurred in older age, but it is now a clinical reality from adolescence onwards [2–4]. Evidence shows a 10-fold increase in the prevalence of T2DM in younger adults and youth over the last couple of decades [5]. This highlights the need for bespoke prevention and treatment pathways that are tailored towards younger populations.

There is now unequivocal evidence that progression to T2DM can be delayed in high risk populations by lifestyle intervention [6]. Traditional diabetes prevention programmes have focused on the promotion of physical activity, a healthy diet, and weight loss and have been shown to reduce the risk of T2DM by 30–60% [6, 7]. However, there is now mounting evidence that these interventions may have overlooked a lifestyle behaviour that is also important to metabolic health, sedentary behaviour. This is defined as any sitting (or lying) behaviours with low energy expenditure during waking hours [7]. Sedentary behaviour is quantitatively different from lack of moderate-to-vigorous intensity physical activity (MVPA). Interventions aimed at increasing MVPA typically focus on promoting behaviour change in a 30–60 minute window, with little regard for what happens during the rest of the day. Therefore, it is possible to meet the current MVPA recommendations for health and still be highly sedentary (i.e. sit a great deal). There is mounting epidemiological evidence that, independent of MVPA, time spent sedentary is associated with a greater risk for all-cause and cardiovascular mortality, chronic disease morbidity and the metabolic syndrome, with strongest effects for cardiometabolic outcomes, particularly type 2 diabetes [8–11]. This observational evidence is supported by experimental research that has shown that regularly breaking sedentary behaviour throughout the day with light activity significantly improves glucose regulation compared to prolonged sitting [12]. Despite the potential beneficial effects of reducing sedentary behaviour on metabolic health, particularly the risk of T2DM, there has been limited research investigating the extent to which this behaviour can be modified using established behaviour change programmes [13–15].

There is now a plethora of evidence for the effectiveness and feasibility of group-based structured education programmes in people diagnosed with, or at high risk of, T2DM, but none have these have addressed younger adults, nor sedentary behaviour. Many organisations [16] highlight and recommend the use of structured education as a means of empowering people with long term conditions. It is also well established within routine primary care [17, 18]. For example, the ‘Diabetes Education and Self-Management for Ongoing and Newly Diagnosed’ (DESMOND) programme has a written curriculum with a person-centred philosophy and learning techniques centred on attendance at one or more face-to-face workshops [19]. Yates et al. [20, 21] successfully used a structured education programme in combination with pedometer use to promote physical activity in those with a high risk of type 2 diabetes through the PREPARE study. The provision of pedometers as a self-monitoring tool seemed to be key in “promoting the self-regulatory strategies needed to convert the motivational impact of the education into sustained behaviour change” [21].

Given the lack of research on younger adults at risk of T2DM at a time when the prevalence of T2DM is increasing in this population, prevention strategies for this age group require development and testing, including targeting sedentary behaviour. Research has shown that reductions in sitting time can be achieved in the workplace [15], often through the use of sit-stand desks [22]. However, the use of other behaviour change strategies, such as counselling, is less clear [23].

We aimed to investigate, through a randomised controlled trial design, whether a group-based structured education workshop focused on sitting reduction, with self-monitoring, reduced sitting time in this population. We further aimed to see whether favourable changes in key behavioural and glycaemic and metabolic markers of T2DM risk could be achieved.

## Method

A protocol paper has been published with further details of the methods [24] (see S1 File).

### Design

This study was a two-arm, individually randomised controlled trial (RCT). 1:1 randomisation (stratified by age, sex, and ethnicity) was set up by an independent statistician using a computer generated list and was conducted remotely.

### Study population and recruitment

Young adults identified as being at risk of developing T2DM were recruited from primary care in Leicestershire and Northamptonshire, which are areas in central England with a diverse ethnic and socio-economic makeup. Invitations were sent from the General Practitioner to potential participants. Inclusion criteria were:

1. Age 18–40 years inclusive
2. BMI in the obese range (≥30kg/m^2^ with ≥27.5kg/m^2^ for South Asians) or BMI in the overweight range (≥25kg/m^2^ with ≥23kg/m^2^ for South Asians) and with one or more additional risk factor for diabetes from i). family history of diabetes or cardiovascular disease in a first degree relative; ii). previous gestational diabetes; iii). polycystic ovarian syndrome; iv). HbA1c ≥5.8%; v). impaired glucose tolerance and/or impaired fasting glucose [25].

We anticipated that obese and overweight 18–40 year olds would be a hard to reach group and as such we provided participants with 20 pounds sterling for each clinic visit in addition to reimbursing travel expenses. The study was approved by the Nottingham National Health Service Research Ethics Committee in May 2010 (see S1 Protocol). All participants signed written informed consent. The trial was registered on 22nd Feb 2011 but started on 17^th^ November, 2010 as this included preliminary qualitative work with participants not included in the RCT [24]. The first participant was consented for the RCT on 9^th^ March, 2011and the last participant was seen for their 12 month follow up visit on 23^rd^ October, 2012. The authors confirm that there are no ongoing or related trials for this intervention.

### The Intervention and control group

Participants attended the baseline study visit and were then randomised to either the control or intervention arm. The control group received an information leaflet focusing on key illness perceptions of being at risk of T2DM, the importance of increasing physical activity and decreasing sedentary behaviour. Each individual in the intervention arm was invited to attend a single 3-hour group-based structured education workshop delivered by two trained educators aimed at targeting knowledge and perceptions of prevalent risk factors for type 2 diabetes and promoting sedentary behaviour change. The workshop was based on previous structured education programmes [20, 26]. A rationale and detailed outline of the 3-hour workshop has been reported previously [24]. In addition to attending the workshop, participants in the intervention arm were given a sedentary behaviour and physical activity self-monitoring device to aid behaviour change (‘Gruve’; MUVE, Inc, USA: http://www.gruvetechnologies.com/) [24] and received a follow-up phone call six weeks after their attendance at the workshop. This was to review their progress, and to discuss their goals and barriers with the aim of supporting behaviour change. This is separate from the research-grade assessment devices used for outcomes measurement and was used for self-monitoring only. The usefulness of the Gruve device was also discussed by phone.

Blinding participants to groups is not possible in this type of study. This could be a source of bias and a limitation.

### Outcome measures

All primary and secondary outcome measures were recorded at study visits at 0, 3 and 12 months. The primary outcome was a reduction in sedentary behaviour at 12 months, measured using the Actigraph GT3X accelerometer. Sedentary behaviour was defined as <100 counts per minute [27].

Participants were requested to wear the accelerometer on a waistband (in the right anterior auxiliary line) for ten consecutive days during waking hours. The Actigraph was initialised with a start and stop time and a 5 second epoch. Data were processed using a commercially available tool (KineSoft version 3.3.76, Kinesoft, Loughborough, UK; www.kinesoft.org). A ‘valid day’ consisted of at least 10 hours of accelerometer movement data and participants with less than 4 days of valid wear were excluded from the analysis. Non-wear time was defined as 60 minutes of continuous strings of zero counts.

### Secondary outcomes

#### Sedentary and physical activity measures

We assessed physical activity and posture allocation as secondary outcomes using both objective monitors and self-report methods. The Actigraph GT3X accelerometer assessed total body movement (counts per day), and time in light-, moderate- and vigorous-intensity physical activity as determined by counts per minute using Freedson cut points [28]. An activPAL3^™^ was worn for the same 10 day period as the ActiGraph and participants were asked to wear the device continuously 24 hours/day. The device was initialised using manufacturer’s software with the default settings (i.e., 20Hz, 10s minimum sitting-upright period) and was covered in a nitrile sleeve and fully wrapped in waterproof dressing (Hypafix Transparent) to allow participants to wear the device during bathing activities. Participants wore the activPAL3^™^ on the midline anterior aspect of the upper thigh and secured it using hypoallergenic waterproof dressing (Hypafix Transparent). To isolate waking wear hours from ‘sleeping’ (i.e., time in bed), prolonged non-wear periods and invalid data, an automated process developed in STATA v13 was applied to event files [29]. Outcome variables included time spent sitting/lying, standing, stepping, and number of sitting/lying to upright transitions.

Self-reported assessment of physical activity and sitting time was made using the short form of the International Physical Activity Questionnaire (IPAQ) [30]. The short ‘last 7 days’ self-administered format was used. Total and Domain-Specific Sitting Questionnaire [31] was used to provide greater contextual information. The scale comprises five items to assess time spent sitting (hours and minutes) each day while (a). travelling to and from places (e.g., work), (b). at work, (c). watching television (TV), (d). using a computer at home, and (e). in leisure time but excluding watching TV (e.g., dining out), on a weekday and a weekend day.

#### Biochemical variables

Participants were invited to attend each clinical measurement session after a 12-hour fast and 24 hours of avoiding vigorous intensity exercise. Glucose control and insulin sensitivity were assessed using standard laboratory methodology for fasting glucose, 2-hour post challenge glucose, fasting insulin, and HbA1c. Serum total cholesterol, HDL cholesterol, triglycerides were also measured. Low density lipoprotein cholesterol (LDL) was estimated using the Friedewald equation [32].

#### Anthropometric, demographic and psychological data

Arterial blood pressure, body weight, body fat percentage, waist circumference and height were recorded. Information on current smoking status, medical and medication history, family history and ethnicity were obtained by self-report.

Several important psychological variables were assessed to establish whether any intervention effect is mediated by the targeted theoretical constructs or whether important psychosocial outcomes are obtained. Data collected included quality of life (EQ-5D), self-efficacy for sedentary behaviour change, and anxiety and depression using the Hospital Anxiety and Depression Scale ([24]).

### Sample size

The minimum reduction in sedentary behaviour which would yield beneficial metabolic effects has not been determined. Cross-sectional data suggests that a 10% increase in sedentary time is associated with a 3.1cm increase in waist circumference, and that sedentary time is positively associated with clustered metabolic risk [27]. Using the same dataset, the mean sedentary time is 56.7 hours/week (8.1 hours/day). Assuming a minimum clinically important difference of 10% (5.67 hours/week) and a standard deviation of 12.1hours/week [27], we required 72 individuals to complete the study per arm assuming an alpha of 0.05 and 80% power. Target recruitment was set at 90 individuals per arm to allow for an estimated dropout rate of 20%.

### Data analysis

The study is reported according to the CONSORT statement for randomised controlled trials (see S1 CONSORT Checklist). Primary analyses were on an intention-to-treat (ITT) basis. Randomisation groups were compared at 3 and 12 months using regression analysis adjusted for the baseline value and stratification factors: age (<28, ≥28 years), sex (male, female) and ethnicity (white, non-white). Missing baseline values for outcomes were replaced with mean values across the whole study, not by arm. Missing outcomes were replaced using multiple imputation. Sensitivity analyses were performed for the 3 and 12 month outcomes by performing per-protocol analyses of those who attended the intervention workshops, and complete case analyses, that is missing data were not imputed. To limit multiple testing, per-protocol and complete case analyses were only performed for the sedentary outcomes at the time of the primary end-point (12 months). Sensitivity analyses were also conducted using two different accelerometer cut-points (<50 and <150 counts/min). Analyses were performed in STATA v13. All p-values are two-sided. The statistician who analysed the data was blinded to treatment allocation.

## Results

### Participants

**Fig 1:**
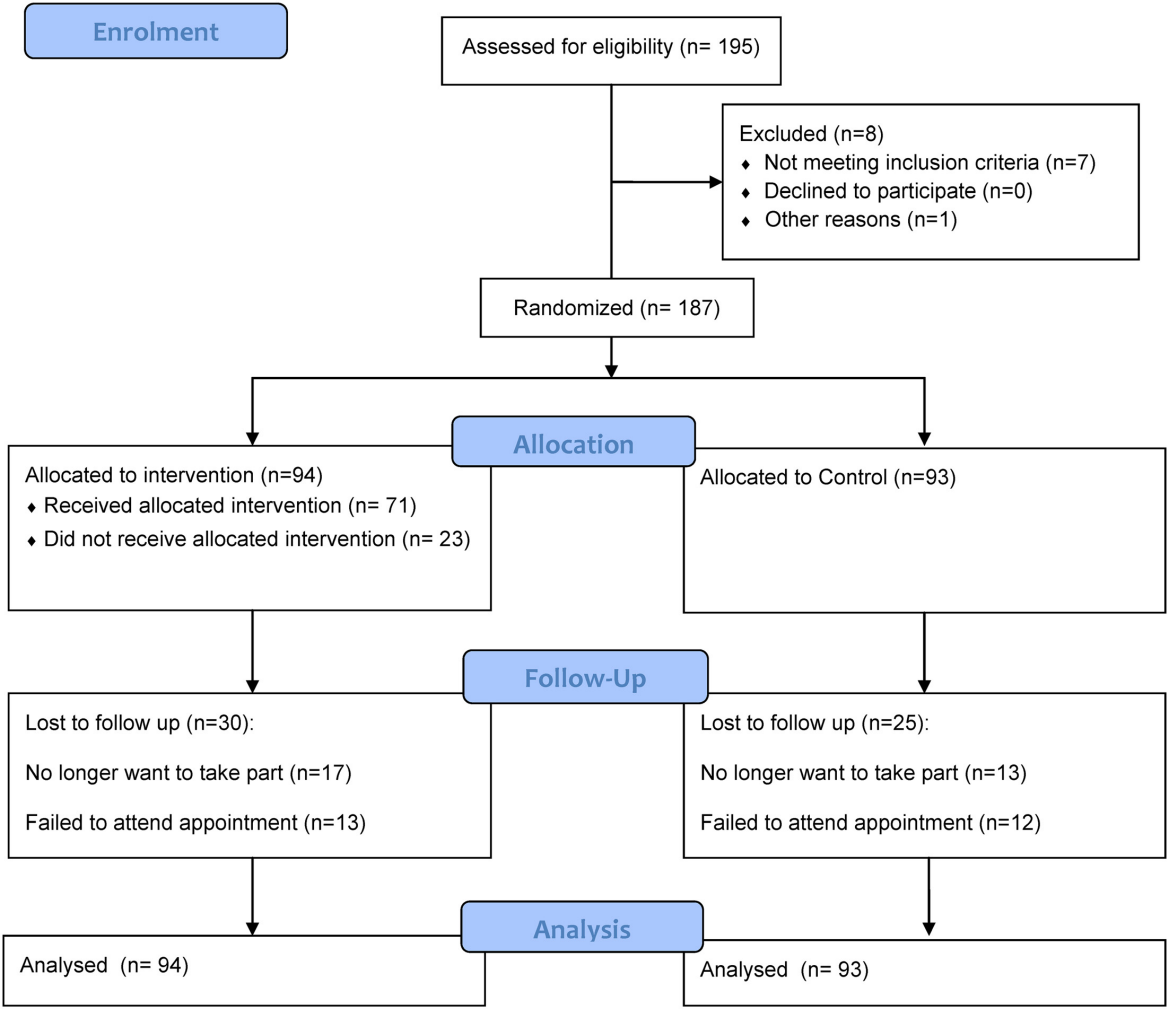
CONSORT diagram of participant flow through the trial.

The trial profile, using the CONSORT guidelines [33], shows participant progress (see Fig 1). Recruitment started in 2011 and the final participant was assessed in 2012. Table 1 provides descriptive statistics for intervention and control groups. Random assignment led to the control group being slightly older and having fewer females than the intervention group, but these differences were small. We were successful in recruiting a clearly at-risk group, with a mean BMI of 35 kg/m^2^ and 85% being obese. Moreover, the sample was highly sedentary, with both groups having mean values around 11 hours per day.

**Table 1:** Descriptive characteristics at baseline by randomisation group.

	Group 1 (intervention) (n = 94)		Group 2 (control) (n = 93)		All (n = 187)	
Variable	n	Mean (SD) or %	n	Mean (SD) or %	n	Mean (SD) or %
Age (years)	94	32.4 (5.4)	92	33.3 (5.8)	187	32.8 (5.6)
Gender (% female)	94	70.2	93	66.7	187	68.5
Ethnicity (% black and minority ethnic group)	93	19.4	93	20.4	187	19.8
Systolic blood pressure (mmHg)	94	118.6 (12.8)	93	121.7 (14.2)	187	120.1 (13.6)
Diastolic blood pressure (mmHg)	94	82.5 (8.6)	93	84.8 (10.4)	187	83.6 (9.6)
BMI (kg/m^2^)	94	34.6 (4.9)	93	34.5 (5.0)	187	34.6 (4.9)
Obese (%)	94	86.2	93	82.8	187	84.5
Waist (cm)	94	103.9 (13.8)	93	102.7 (14.0)	187	103.3 (13.9)
Body fat (%)	94	40.8 (7.2)	93	40.4 (7.0)	187	40.6 (7.1)
Fat-free mass (%)	93	57.2 (13.8)	93	57.7 (12.4)	186	57.4 (13.1)
Cholesterol (mmol/l)	94	4.9 (0.9)	92	5.0 (1.0)	186	4.9 (1.0)
LDL (mmol/l)	93	2.9 (0.8)	89	3.0 (0.8)	182	3.0 (0.8)
HDL (mmol/l)	94	1.2 (0.3)	91	1.3 (0.3)	185	1.3 (0.3)
Triglycerides (mmol/l)	94	1.5 (0.8)	92	1.6 (1.5)	186	1.5 (1.2)
HbA1c (%)	93	5.6 (0.4)	92	5.6 (0.3)	185	5.6 (0.3)
Fasting glucose (mmol/l)	93	4.8 (0.6)	93	4.8 (0.5)	187	4.8 (0.5)
2-h glucose (mmol/l)	94	5.4 (1.8)	92	5.4 (1.4)	185	5.4 (1.6)

In total 30 (32%) in the intervention group and 25 (27%) in the control group were lost to follow-up at 12 months (see S1 Table). Those who were lost to follow-up in the intervention group were not significantly different on any characteristics from those who completed the study. Those who were lost to follow-up in the control group tended to live in areas with a higher multiple deprivation score and to be unemployed (control group only). Of those allocated to the intervention, 23 (24%) did not attend the structured education workshop. These individuals were more likely to be in employment (see S2 Table).

### Primary outcome

The primary outcome variable was accelerometer-determined sedentary time at 12 months. Table 2 shows that small non-significant reductions were evident at 12 months for both groups when analysing data using ITT. The intervention group reduced daily sedentary time by 17.4 minutes per day (95% CI: 45.0 mins/day decrease, 10.2 mins/day increase) and the control group by 13.8 minutes (95% CI: 36.0 mins/day decrease, 8.4 mins/day increase). The adjusted difference between the change in the two groups was not significant (p = 0.52). Sensitivity analyses revealed that the results were not affected by using alternative cut-points of <50 counts/min and <150 counts/min.

**Table 2:** Sedentary outcomes by randomisation group (intention to treat analysis)^a^.

	Group 1 (Intervention)		Group 2 (Control)			
Outcome measure	n	Mean (95% CI)	n	Mean(95% CI)	Difference (95% CI)	P-value
**Accelerometer measures**						
Average sedentary time per day, hours^b^						
Baseline	76	10.83 (10.50, 11.17)	80	11.01 (10.76, 11.26)		
Change at 3 months	45	-0.08 (-0.48, 0.32)	54	-0.13 (-0.45, 0.19)	0.01 (-0.49, 0.52)	0.956
Change at 12 months	38	-0.29 (-0.75, 0.17)	49	-0.23 (-0.60, 0.14)	-0.19 (-0.80, 0.41)	0.519
Average number of breaks in sedentary behaviour per day (LVPA bouts)						
Baseline	76	694.7 (657.2, 732.2)	80	672.3 (639.9, 704.7)		
Change at 3 months	45	-31.1 (-70.3, 8.01)	54	18.3 (-20.5, 57.1)	-29.6 (-97.0, 37.9)	0.383
Change at 12 months	38	-1.92 (-42.8, 39.0)	49	9.56 (-39.9, 59.0)	-2.96 (-73.0, 67.0)	0.932
**ActivPal measures**						
Average sedentary time per day, hours						
Baseline	60	8.91 (8.59, 9.24)	57	9.02 (8.73, 9.30)		
Change at 3 months	34	0.75 (0.28, 1.21)	35	0.60 (0.09, 1.11)	0.09 (-0.55, 0.72)	0.785
Change at 12 months	32	0.64 (0.13, 1.16)	29	0.58 (0.06, 1.09)	-0.12 (-0.99, 0.76)	0.789
Total sedentary to upright movements						
Baseline	60	53.4 (50.6, 56.1)	57	51.9 (49.9, 53.9)		
Change at 3 months	34	19.1 (14.3, 24.0)	35	20.0 (14.7, 25.3)	-2.14 (-10.42, 6.14)	0.607
Change at 12 months	32	7.96 (3.29, 12.6)	29	5.63 (0.50, 10.76)	-0.19 (-6.99, 6.61)	0.955

^a^ Adjusted for stratification factors. For accelerometer and activPAL variables, additionally adjusted for change in waking wear time.

^b^ Primary outcome.

### Secondary outcomes

Generally, there were no significant changes at 12 months in the other objectively measured (Table 2) and self-reported (S3 Table) measures of sedentary behaviour although the intervention group did show a significant reduction in self-reported sitting.

Data for physical activity, as assessed by the Actigraph accelerometer, activPAL, and self-report, as well as biochemical, anthropometric, and psycho-social variables is shown in S4 Table. At 12 months, there were no significant changes in any of the objectively assessed or self-reported physical activity variables. For the biochemical, anthropometric and psycho-social variables, no significant differences were noted at 12 months.

### Sensitivity analyses

Results were unchanged in the per-protocol analyses and complete cases analysis (see S5 Table).

## Discussion

A randomised controlled trial using structured education and self-monitoring failed to show a significant reduction in sedentary behaviour in young adults at risk of T2DM when compared to controls. As far as we are aware this is the first RCT to address changes in sedentary behaviour for this age group and at risk of T2DM population. Results are in contrast to other group-based structured education interventions with older adults targeting lifestyle change [26] and physical activity [21]. However, in the study by Davies et al. [26], the workshops were for 6 hours—twice the time allocated to the current RCT. They found significant favourable changes in biomedical, psychosocial, and lifestyle measures in adults (mean age 60 years) with newly diagnosed type 2 diabetes. However, in the physical activity RCT reported by Yates et al. [21], with adults above an average age of 64 years and with impaired glucose tolerance, only the group receiving 3 hours of structured education and self-monitoring, through the use of pedometers, increased their physical activity.

This suggests that our 3 hour structured education workshop may not be enough to achieve behaviour change in young adults at risk of T2DM. Moreover, structured education for this group may also require successful use of self-monitoring—something we felt we did not achieve across all participants (see later).

It is possible that a one-off educational approach with self-monitoring, even when based on prior experience and using a patient-centred approach, is simply not potent enough to bring about sedentary behaviour change. This may be exacerbated by the population recruited. The population targeted was younger adults at risk of T2DM. Adults in the range of 18–40 years are likely to perceive little immediate risk, especially associated with a ubiquitous behaviour like sitting.

Our results are consistent with two related studies. Evans et al’s [13] study of office workers involved education concerning the consequences of prolonged sitting in addition to one group also receiving prompts on their computer to break up their sitting. Results showed that the education-only group did not change. However, sitting was reduced in the education plus prompts group. Similarly, in an intervention reported by Aadahl et al. [23], with men and women with a mean age in their early 50s, the use of motivational counselling was not successful in significantly reducing sitting time in comparison to controls.

The structured education in our study allowed participants to learn more about diabetes and sedentary behaviour, as well as explore possible ways to reduce their sitting time. However, once they left the workshop, they were essentially on their own to embed the strategies in to their lives. Behaviour change science is now suggesting that education is just one of many ways that could change behaviour [34, 35]. In addition, motivation to change will involve conscious decision making as well as less conscious ‘automatic’ processes. The latter involve acting in accordance with basic likes and dislikes and with rather little deliberation. Whether it is possible to create a situation where not sitting (i.e., standing and other light movement) is seen as ‘pleasurable’, and hence the default option, has not been tested. We also need to investigate more overt changes to the environment. Automatic processing will involve habitual reactions to the environment and acting out of habit. This is highly likely for sedentary behaviour where chairs are provided and sitting is the norm. Provision of sit-stand desks, for example, has been shown to successfully reduce sitting time [15, 22]. In short, it appears that education may not be enough to reduced sedentary behaviour and that environmental changes may be needed. This may particularly be the case if participants do not see themselves at high risk of diabetes in the first place. Process evaluation (not reported here) suggested that this may have been the case for many participants.

Feedback from participants also suggested that many participants tried to increase their physical activity rather than reduce their sitting time. This may have been associated with higher visibility in the media about physical activity and the confusion that can occur between ‘being sedentary’ (i.e. sitting) and ‘being inactive’ (i.e. not participating in moderate-to-vigorous physical activity or ‘exercise’). In a recent meta-analysis, it was shown that interventions focussing on physical activity—alone or in combination with sedentary behaviour—showed no evidence of a statistically significant effect for reducing sedentary behaviour [36].

One element of our intervention was the use of a self-monitoring device. In addition to allowing feedback on sedentary time (via a computer), this prompted the participant through its vibration function if they had been sitting for an extended period. Research suggests that self-monitoring is an important and successful behaviour change technique [37]. Feedback from participants yielded mixed views about the self-monitoring device, although many were positive. Logistical difficulties were mentioned and the device did not allow real-time feedback, other than the prompt function. With the rapid development of wearable technologies, and given that the Gruve was selected for the study in 2010, it is clear that newer technologies will have better functionality and provide real-time feedback. This should increase the probability of such a device facilitating behaviour change.

One key factor that requires attention in future trials is that of non-attendance at the structured education workshop. This was quite high at 25% and this seemed to be a function of being in work. Sessions were offered and run outside of normal working hours, but achieving good attendance was still challenging. The participant burden of attending a 3h structured educational workshop is high. Moreover, we had high attrition at follow up visits and we underestimated dropout and non-compliance with the primary outcome measure. This led to large amounts of missing data. Future studies working with this type of population need to address this and be aware of the challenges of recruiting and retaining such participants. More needs to be known about the demands of attending an educational workshop and testing sessions, and the consequences of being asked to wear certain types of movement sensors throughout the day.

In summary, we were not successful in bringing about a reduction in sedentary behaviour at 12 months for young adults at risk of T2DM. Future trials should consider the nature and length of structured education for this age group, how to include environmental changes, making a clear distinction between ‘too much sitting’ and ‘too little exercise’, and better technologies to self-monitor and prompt behaviour change. The ubiquitous and habitual nature of sitting in modern society may mean that behaviour change will be challenging.

## Supporting Information

S1 CONSORT Checklist CONSORT checklist. (DOC)

S1 File Protocol published paper. (PDF)

S1 Protocol Trial Protocol. (DOC)

S1 Table Characteristics of those who did and did not complete the study, by treatment group. (DOCX)

S2 Table Characteristics of those in intervention group who attended/did not attend education session. (DOCX)

S3 Table Self-reported sedentary behaviours. (DOCX)

S4 Table Secondary outcomes by randomisation group. (DOCX)

S5 Table Sensitivity analysis. (DOCX)
