# Supplementary material for: A Randomised Controlled Trial to Reduce Sedentary Time in Young Adults at Risk of Type 2 Diabetes Mellitus: Project STAND (Sedentary Time ANd Diabetes)
Source: PLoS One. 2015 Dec 1;10(12):e0143398. doi: 10.1371/journal.pone.0143398 (PMC4666612; doi:10.1371/journal.pone.0143398)
Supplement: S1 File — (PDF) [file pone.0143398.s002.pdf]

Wilmot et al. BMC Public Health 2011, 11:908
http://www.biomedcentral.com/1471-2458/11/908

BMC Public Health

STUDY PROTOCOL Open Access

# Rationale and study design for a randomised controlled trial to reduce sedentary time in adults at risk of type 2 diabetes mellitus: project STAND (*Sedentary Time ANd Diabetes*)

Emma G Wilmot[1], Melanie J Davies[1], Charlotte L Edwardson[2,3], Trish Gorely[3], Kamlesh Khunti[4], Myra Nimmo[3], Thomas Yates[1] and Stuart JH Biddle[3*]

## Abstract

**Background:** The rising prevalence of Type 2 Diabetes Mellitus (T2DM) is a major public health problem. There is an urgent need for effective lifestyle interventions to prevent the development of T2DM. Sedentary behaviour (sitting time) has recently been identified as a risk factor for diabetes, often independent of the time spent in moderate-to-vigorous physical activity. Project STAND (*Sedentary Time ANd Diabetes*) is a study which aims to reduce sedentary behaviour in younger adults at high risk of T2DM.

**Methods/Design:** A reduction in sedentary time is targeted using theory driven group structured education. The STAND programme is subject to piloting and process evaluation in line with the MRC framework for complex interventions. Participants are encouraged to self-monitor and self-regulate their behaviour. The intervention is being assessed in a randomised controlled trial with 12 month follow up. Inclusion criteria are a) aged 18-40 years with a BMI in the obese range; b) 18-40 years with a BMI in the overweight range plus an additional risk factor for T2DM. Participants are randomised to the intervention (n = 89) or control (n = 89) arm. The primary outcome is a reduction in sedentary behaviour at 12 months as measured by an accelerometer (count < 100/min). Secondary outcomes include physical activity, sitting/lying time using the ActivPAL posture monitor, fasting and 2 h oral glucose tolerance test, lipids, inflammatory biomarkers, body weight, waist circumference, blood pressure, illness perceptions, and efficacy beliefs for behaviour change.

**Conclusions:** This is the first UK trial to address sedentary behaviour change in a population of younger adults at risk of T2DM. The results will provide a platform for the development of a range of future multidisciplinary interventions in this rapidly expanding high-risk population.

**Trial registration:** Current controlled trials ISRCTN08434554, MRC project 91409.

## Background

There has been a dramatic increase in the incidence of Type 2 Diabetes Mellitus (T2DM). Until recently T2DM was considered a disease of older adults, however, this condition is now diagnosed in children and young adults and between 1998 and 2005 there was an eightfold increase in the prevalence of T2DM in young people in the UK [1]. Younger adults with T2DM are more likely to be obese, have a strong family history of T2DM, lead a sedentary lifestyle, be of black or minority ethnic (BME) origin, and come from less affluent socio-economic groups [2-4]. At the time of diagnosis, one in five young people with T2DM have evidence of abnormal kidney function, four in five have abnormal lipids and half have hypertension [5]. These risk factors translate into a substantially increased risk of morbidity and mortality; for example the hazard of developing a myocardial infarct in early-onset T2DM (< 45 years) is 4-fold higher

* Correspondence: S.J.H.Biddle@Lboro.ac.uk
[3]School of Sport, Exercise and Health Sciences, Loughborough University, Loughborough LE11 3TU, Leicestershire, UK
Full list of author information is available at the end of the article

BioMed Central

© 2011 Wilmot et al; licensee BioMed Central Ltd. This is an Open Access article distributed under the terms of the Creative Commons Attribution License (http://creativecommons.org/licenses/by/2.0), which permits unrestricted use, distribution, and reproduction in any medium, provided the original work is properly cited.

than in late onset T2DM (> 45 years) and 14-fold higher than in people without T2DM [6]. From a societal perspective, the explosion of young people developing T2DM has significant implications for future workforce and health care systems. It is crucial that effective lifestyle interventions are urgently developed and employed to prevent the development of diabetes in younger at-risk groups.

Sedentary behaviour has recently been proposed as a key driver of the current obesity and diabetes epidemics, often independent of other related behaviours such as physical activity [7]. The term 'sedentary' comes from the latin *sedre* ('to sit') and can operationally be defined as sitting or lying with very low energy expenditure [8]. The opportunities for sedentary behaviours, such as watching television, sitting in a car or using the computer, are ubiquitous. It is known from objective measures of activity that adults spend 50-60% of their waking day in sedentary pursuits [9], and evidence is accumulating which suggests that excess sedentary time has a negative impact on health, often independent of the time spent in moderate to vigorous activity [7].

The hazards of sitting were first highlighted in the 1950's when Jeremy Morris identified a two fold increase in the risk of a myocardial infarction in London bus drivers compared with active bus conductors [10]. In the past decade the interest in the study of sedentary behaviours has been reignited, with the publication of a range of observational data reporting associations between sedentary time and adverse health outcomes [11], including an increased risk of diabetes, cardiovascular and all-cause mortality [12-14].

The STAND structured education randomised controlled trial was designed to assess whether reducing sedentary time was possible, and if so, what the associated health benefits were in young adults with a high risk of T2DM.

### Rationale for the STAND intervention

Structured patient education forms the core of the STAND intervention and is based on the DESMOND self-management protocol [15] and related interventions such as PREPARE [16] and are consistent with NICE guidance [17]. Structured education programmes encourage patients to participate in an active way in their learning about diabetes and associated risk behaviours, usually through non-didactic led educational workshops that include group discussions, experiential learning and practice, self-monitoring and goal setting to promote self-efficacy and behaviour change [18]. To this end, it is important to base an intervention on sound behavioural theories [19]. This is explained in the section on 'Methods (Phase 2)' below.

The DESMOND and PREPARE programmes were designed for the older adult and did not take into account the specific issues facing younger adults. There was a need to develop an effective lifestyle self management programme for younger adults at risk of T2DM, incorporating the emerging evidence on sedentary behaviour and its associated negative health outcomes. The STAND education programme, therefore, was developed through modifications of existing programmes to focus on reducing sedentary behaviour and the specific needs of younger adults.

## Methods

Project STAND encompasses three distinctive phases which were informed by the MRC framework for complex interventions [20]. Phases 1 and 2 comprise qualitative data collection and analysis leading to the development and piloting of an evidence based structured self-management education programme. Phase 3 is the delivery of the STAND randomised controlled education and lifestyle intervention trial. This is a 2-arm parallel randomised controlled trial (RCT), with 12 month follow up, to compare the effectiveness of structured education and self monitoring (intervention) with usual care (control arm).

### Phase 1: Qualitative data collection and analysis

Semi-structured interviews were conducted to inform the development of the STAND programme. Views and perceptions of T2DM, awareness and acceptability of reducing sedentary behaviour and opinions about educational interventions were discussed with 14 overweight or obese young adults aged 18-40 years with at least 1 risk factor for T2DM, thus being representative of the sample to be recruited for the main trial. Data saturation was thought to be achieved, hence the sample size of 14. Interviews were transcribed and a thematic analysis was conducted to identify key themes. Ethical approval was granted by the Loughborough University Ethical Advisory Committee.

Participants demonstrated limited knowledge of T2DM and awareness of risk factors was limited to general comments on 'diet' and 'exercise'. Most viewed T2DM as something that happened later in life and it appeared to have little personal meaning to them. All participants stated that they would try to change if they were told they were at high risk but less than half actually recognised that they were at risk. Reducing sedentary behaviour, while new to them, was seen as something they were willing to attempt, although when and where this would be possible varied between individuals. Group based education would be acceptable but it would need to be approached and presented in a way to maximise attendance among those most in need.

From the qualitative work it was clear that creating a personal understanding of the risk factors for, and consequences of, diabetes, in keeping with Leventhal's Common Sense Model [21], would be vital to the success of the intervention.

### Phase 2: Curriculum development

The qualitative findings were used to adapt the PREPARE and DESMOND structured education programmes to target a reduction in sedentary behaviour in young adults. Modifications to curricula and the development of visual aids were overseen by investigators with expertise in health behaviour theory, physical activity and sedentary behaviour, diabetologists, diabetes specialist nurses, a general practitioner and a dietician. A written curriculum was developed to incorporate the key psychological theories (see below for detail). For example, behaviour change was encouraged through the use of self-regulatory strategies, by increasing the confidence of subjects to reduce sedentary behaviour, and by encouraging their awareness of how to make less sedentary options more attractive and available, as well as limiting time in some sedentary pursuits in line with Social Cognitive Theory and Behavioural Choice theories.

Both the DESMOND and PREPARE education programmes combined several mutually supportive theories at its core, including Bandura's Social Cognitive Theory, focusing on the central concepts of self-efficacy (confidence to undertake the behaviour), targeting barriers and self regulating behaviour [22]. Self regulation is key to successful health behaviour change and as such was a key component of the STAND intervention [23]. Furthermore, Gollwitzer's [24] implementation intentions concept was another important framework for the development of successful strategies around self-regulation such as focusing on the where, when and how of planned behaviour in order to close the gap between intention and behaviour [24]. Closely linked to these theories was Behavioural Choice Theory which postulated that behavioural choices were the result of the accessibility of the behaviour and its reinforcement value [25].

Perceptions of risk and health beliefs are also important. Leventhal's Common Sense Model postulates that individuals conceptualize identified health threats in terms of the cause, consequences, identity, control/treatment and the timeline associated with the threat and that these domains influence subsequent coping behaviour [21]. These illness perceptions and beliefs have been closely linked to health behaviour change in individuals with T2DM [26].

Self monitoring is key to the success of behaviour change interventions [22,23]. The study team researched and piloted a number of potential devices to facilitate sedentary behaviour self-monitoring. An objective measure of sedentary time was sought which would provide dynamic feedback for participants. The study of sedentary behaviour is still developing and three devices were identified at the time and which were available to pilot: PAM Coach (Move2Health, The Netherlands: http://www.pam.com), ActivPAL (PAL Technologies Ltd., Glasgow, UK: http://www.paltechnologies.com), and Gruve (MUVE, Inc., USA: http://www.muveinc.com/gruve.asp) devices. The PAM device measures different levels of activity and converts them into 'PAM points'. However, feedback from pilot testing showed that the device was not particularly intuitive to use and importantly, it did not present the amount of time spent sedentary for self monitoring. The ActivPAL is a thigh worn device which determines posture on the basis of thigh inclination and using proprietary algorithms (Intelligent Activity Classification) classifies activity into time spent sitting/lying, standing, or stepping. While the feedback from this device provided a reliable estimate of time spent sitting, using this device would require the participants to regularly initialise their monitor and download their data using expensive specialised equipment and software. Moreover, self-monitoring is encouraged for as long as the participants wish during the trial. Feedback suggests that wearing the ActivPAL taped to the thigh can be uncomfortable beyond about 1 week. It was concluded that the activPAL was more appropriate as a tool for researchers rather than a self-monitoring tool for participants. Nonetheless, the team recognised the potential of the ActivPAL and opted to incorporate its sedentary time output in the trial as a secondary outcome measure. However, in the educational workshop, personalised data from the ActivPAL are presented to participants. This was achieved by meeting the participants at least 1 week prior to the workshop to instruct them on how to use the device. Data used from the ActivPAL includes their total sitting time as well as a breakdown of their sitting patterns throughout the day. Long and short-term goals are then set based on this information, such as targeting less sitting at times of the day where sitting is high and seen to be acceptable to change.

The Gruve device is a waist worn accelerometer which monitors sedentary time and data are downloaded easily to the interactive Gruve website. This enables the participants to view and track progress on time spent sedentary. Time spent sedentary can be viewed on daily, weekly and monthly data charts, allowing the participant to set and revise personal goals. Furthermore, if the participant is sedentary for a prolonged period the device will vibrate to notify them that they have been sedentary and are reaching their 'energy conservation point' (ECP).

The ECP marks the point at which the body goes into a reduced caloric burn rate following a prolonged period of sedentary behaviour. The frequency of the vibration will vary across participants and depends on the health information inputted by each individual. The vibration function acts as a reminder to stand and move around, providing a helpful prompt for behaviour change. Pilot work with the research team and study participants provided positive feedback on this device and hence was considered the most appropriate self-monitoring device for this study.

### Phase 2: Piloting and modifications

Permission to pilot the education intervention was received from the local primary care trusts. Ethical approval was granted by Nottingham NHS Research Ethics Committee for recruitment from the wider community. Three pilot sessions were delivered–two to participants recruited through advertising materials distributed in the university town of Loughborough, UK, and one to participants recruited from a general practice (GP) nearby. Participants recruited through community recruitment were self selecting. Participants recruited from the GP were identified by a search of GP computer reference systems for those meeting the inclusion criteria followed by an invitation letter from their GP. The structured education course was delivered over one 3-hour session by two educators trained in structured education through the DESMOND collaborative, including training on the modifications required for working with younger adults. Each pilot session was observed by a researcher trained in delivering education sessions and/or qualitative research methodology. The researcher recorded personal observations and conducted semi-structured interviews with participants at the end of the session. Participants also completed feedback forms. The development of the workshop is an iterative process involving pilot work, feedback, revision and further pilot work.

Overall, the pilot education sessions were well received. Participants felt they had greater understanding of diabetes and its risk factors as a result of the course and the visual aids employed. Some participants had suggestions for improvements to the visual aids which were incorporated prior to the start of the RCT. Participants enjoyed estimating their personal sitting time, comparing this with the objective feedback from the ActivPAL device and subsequently discussing how to reduce sitting time. Participants said that they would like more time for goal planning so we ensured that this was built into the programme. Some felt the benefits of exercise had to be more strongly emphasised in addition to the benefits of standing more/sitting less and, again, this feedback was used to modify the education programme. An overview of the final education intervention can be found in Table 1

### Phase 3: STAND randomised controlled trial

The primary hypothesis of the STAND RCT is that structured education will decrease sedentary behaviour in young adults at risk of T2DM. The secondary hypothesis is that a reduction in sedentary behaviour will result in favourable changes in key behavioural and biological markers of T2DM risk.

### Study population and recruitment

Young adults who are at risk of developing T2DM from across Leicestershire and the South East Midlands Diabetes research network in the UK are recruited (see Figure 1). Inclusion criteria are:

Exclusion criteria include significant illness, steroid use, diabetes, pregnancy or an inability to communicate in English.

Participants are primarily recruited from primary care in Leicester, Northampton and Kettering, areas in England with a diverse ethnic and socio-economic makeup. Recruitment is co-ordinated via the East Midland and South Yorkshire Primary Care Research Network. An electronic GP database search was conducted to identify participants who met the inclusion criteria. Invitations were sent by the GP to the participants who then replied directly to the study team.

Study information is also sent to patients with diabetes who are attending the local hospital, to ask them to pass the study information to relatives aged 18-40 years who might be interested in taking part in the study. We had permission to recruit participants from the wider community and local businesses using posters and media adverts.

We anticipated that obese and overweight 18-40 year olds would be a hard to reach group and as such we provide participants with £20 for each clinic visit in addition to reimbursing travel expenses. All participants will be asked to sign written informed consent.

### Intervention and control arm

Randomisation (stratified by age, sex, and ethnicity) was set up by a independent statistician using a computer generated list and was conducted remotely. Participants attended the baseline study visit and were then randomised to either the control (C) or intervention (I) arm. Participants randomised to the control group received an information leaflet focusing on key illness perceptions of being at risk of T2DM, the importance of increasing physical activity and decreasing sedentary behaviour. Each individual in the intervention arm was invited to attend the STAND structured self-

**Table 1 Potential outline of the STAND structured education course which was delivered to the participants in the intervention arm**

| Module name | Main aims and educator activities | Theoretical underpinning | Time weighting |
|---|---|---|---|
| ***Patient story*** | Participants given opportunities to share their knowledge and perceptions of diabetes risk and highlight any concerns they may want addressed in the programme. | Common Sense Model | 8% (15 min) |
| ***Professional story*** | Simple non-technical language, analogies, visual aids and open questions used to provide participants with an overview of healthy glucose metabolism, the aetiology, risk factors and complications associated with diabetes. Individual feedback provided on biochemical and anthropometric measures measured at baseline visit. Participants encouraged to assess their personal diabetes risk and identify their modifiable risk factors. | Common Sense Model<br>Dual Process Theory | 33% (60 min) |
| ***Sedentary behaviour*** | Simple non-technical language, analogies, visual aids and open questions used to help participants identify the health hazards associated with excess sedentary time and discuss how reducing sedentary behavior may reduce future risk of developing diabetes. Participants provided with printed feedback on their sitting time from the ActivPAL.<br>Participants discussed options for reducing sedentary behaviours in everyday life; identified barriers to reducing sedentary behavior and formed action plans and set personal goals. Practical demonstration of how to use the Gruve device for the self-regulation of sedentary time. | Social Cognitive Theory<br>Behavioural Choice Theory | 58% (105 min) |

management group education programme delivered by trained educators, developed during Phases 1 and 2.

Six weeks after the educational workshop, participants in the intervention arm were contacted by telephone to review progress, discuss goal setting and barriers with the aim of supporting behaviour change maintenance. The usefulness of the Gruve device for self monitoring was also discussed. Text messages were also sent to encourage adherence to the workshop goals and use of the Gruve.

## Sample size/data analysis

The primary outcome is a reduction in sedentary behaviour, measured by an accelerometer at 12 months. The minimum reduction in sedentary behaviour which would yield beneficial metabolic effects has not been determined. Cross-sectional data suggested that a 10% increase in sedentary time is associated with a 3.1 cm increase in waist circumference, and that sedentary time is positively associated with clustered metabolic risk [27]. Using the same dataset, the mean sedentary time is 56.7 h/week. The minimum clinically important difference would be 5.67 h/week, reducing to 51.03 (SD 12.1). Sample size is estimated as 2 N = (4(Za + Zb)2s2)/d2 (where d is the true between-arms difference, b is the type II error rate, and a is the type I error rate). Alpha is set at $P$ = 0.05 (Za = 1.96) and power at 80% (b = 0.20, Zb = 0.842). This results in a required N of 72 in

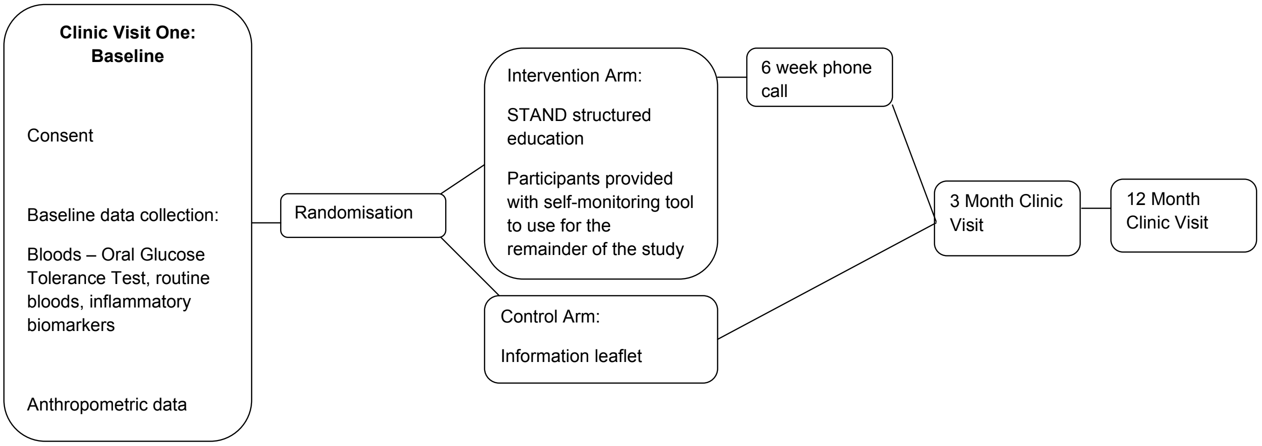


**Figure 1 RCT Flow Chart**. a) Age 18-40 years with a BMI in the obese range (≥ 30 kg/m$^2$; ≥ 27.5 kg/m$^2$ for South Asians). b) Age 18-40 years with a BMI in the overweight range (≥ 25 kg/m$^2$; ≥ 23 kg/m$^2$ for South Asians) and with one or more additional risk factor for diabetes from: • family history of diabetes or cardiovascular disease in a first degree relative; • previous gestational diabetes; • polycystic ovarian syndrome; • HbA1c ≥ 5.8% (from our local Addition Leicester diabetes screening data a cut off HbA1c of 5.8% provided the best sensitivity and specificity for a diagnosis of prediabetes). • Impaired glucose regulation (defined according to the World Health Organisation).

each arm. Incorporating a dropout rate of 20% gives a final N of 89 per arm.

The study is reported according to the CONSORT statement for randomised controlled trials. Data will be analysed on an intention-to-treat basis (ITT). Descriptive statistics (mean values and frequencies) will be calculated. Histograms will be used to identify any outliers and to test for normality. ANCOVA modelling will be used to look at the difference between groups in change in continuous outcome measures, and logistic regression to analyse categorical variables.

### Study measures

All primary and secondary outcome measures are recorded at study visits at 0, 3 and 12 months.

### Primary outcome

The primary outcome is a reduction in sedentary behaviour at 12 months, measured objectively using the triaxial Actigraph GT3X accelerometer. These accelerometers were the most extensively validated and accurate on the market, albeit for physical activity assessment, and they are the only commercially available accelerometers to correlate with energy expenditure as measured by double-labelled water [28]. However, recent studies have also used this device to assess time in sedentary behaviour, although there is still debate about the exact counts per minute to use as a representation of time in sedentary behaviour [29,30]. Accelerometers can provide an estimate of the total volume of sedentary behaviour and are also capable of detecting short, incidental breaks in sedentary time (< 5 min.), which may not be feasibly recorded by self-report measures.

Participants are requested to wear the accelerometer on a waistband (in the right anterior auxiliary line) for ten consecutive days during waking hours. The actiGraph was initialised with a start and stop time and a 5 s epoch. A 'valid day' will consist of at least 10 h of accelerometer movement data and participants with less than 4 days (3 weekdays and 1 weekend day) of valid wear will be excluded from the analysis. Non-wear time will be defined as strings of '0'. The primary outcome measure is sedentary time defined as time < 100 counts per minute [29].

### Secondary outcomes

Physical activity and body posture (sitting, standing, stepping) are measured objectively using the Actigraph and ActivPAL accelerometers as well as through self report using the short International Physical Activity Questionnaire (IPAQ) [31]. The Actigraph GT3X accelerometer is used to measure steps per day, total body movement (counts per day), and time in light-, moderate- and vigorous-intensity physical activity as determined by counts per minute using cut points proposed by Freedson et al. [32]. The ActivPAL is a thigh worn accelerometer and inclinometer which measures the angle of the thigh, providing data on participant posture (i.e. sitting or lying vs standing) and time spent in sedentary behaviour (sitting or lying). The activPAL has been shown to be a valid and reliable tool for the assessment of sitting in adults [33-35]. The ActivPAL is worn on the thigh for the same 10 day period as the accelerometer. The short 'last-seven-days' self-administered format of the IPAQ is used as a self-report measure of physical activity and sitting time. This questionnaire provides a comprehensive measure of moderate- to vigorous-intensity activities carried out for more than 10 continuous minutes at work, in the home, as transport and during leisure time. IPAQ has been shown to have reasonable validity compared to accelerometer data ($\rho \sim 0.4$) and test-retest reliability ($\rho \sim 0.7$) in the UK when used as a measure of total moderate- to vigorous-intensity physical activity [36]. The reliability and validity of the IPAQ sitting questions in a sample from four countries were acceptable, with validity tested against accelerometers [31].

### Biochemical variables

Participants are invited to attend each clinical measurement session after a 12-hour fast and 24 h of avoiding vigorous intensity exercise. Glucose control and insulin sensitivity are assessed using standard laboratory methodology for fasting glucose, 2-hour post challenge glucose, fasting insulin, and HbA1c. Serum total cholesterol, HDL cholesterol, and triglycerides; liver function, urea and creatinine and vitamin D will be measured. Low density lipoprotein cholesterol (LDL) is estimated using the Friedewald equation [37]. Serum is collected and frozen for subsequent analysis of inflammatory bio-markers (hsCRP, TNF alpha, sIL-6, and sIL-6R) and stored until complete sample sets are collected for a participant when all time points are assayed to avoid any intra-assay variation (instructions for freezer samples available on request).

### Anthropometric, demographic and psychological data

Arterial blood pressure is measured in the sitting position (Omron, Healthcare, Henfield, UK); three measurements are obtained and the average of the last two measurements are used. Other measures include body weight and body fat percentage (Tanita BC 420SMA, Tanita, West Drayton, UK), waist circumference (midpoint between the lower costal margin and iliac crest), and height to the nearest 0.1 kg, 0.5% and 0.5 cm respectively. Information on current smoking status, medical and medication history, family history and ethnicity are obtained by self-report.

Several important psychological variables are measured to establish whether any intervention effect is mediated by the targeted theoretical constructs or whether important psychosocial outcomes are obtained. Data collected include quality of life [38], illness perceptions using the Brief Illness Perceptions Questionnaire [39], self efficacy [40], fatigue and sleep [41,42], and anxiety and depression using the Hospital Anxiety and Depression Scale [43].

## Conclusion

Project STAND is the first UK trial, to our knowledge, to address sedentary behaviour change in a population of young adults at risk of T2DM. The trial informs behaviour change programmes for at-risk groups, and provides a major new direction of behaviour change alongside the more conventional approach of encouraging increases in moderate-to-vigorous physical activity. The results will provide a platform for the development of a range of future multidisciplinary interventions in this rapidly expanding high-risk population.

### Abbreviations

BMI: Body mass index; DESMOND: Diabetes education and self management for ongoing and newly diagnosed; GP: General practice/general practitioner; IPAQ: International Physical Activity Questionnaire; LPL: Lipoprotein lipase; MRC: Medical Research Council; MRI: Magnetic resonance imaging; PREPARE: Pre-diabetes risk education and physical activity recommendation and encouragement; RCT: Randomised controlled trial; STAND: Sedentary time and diabetes; T2DM: Type 2 diabetes mellitus.

### Acknowledgements

We would like to acknowledge the following people who have collected data or contributed to the running of the study:Jeanette Barnett, Paul Bray, Jane Brela, Lesley Bryant, Heather Daly, Jayne Hill, Janet Jarvis, Champa Merry, Mo Radia, Jason Rigby, Lorraine Martin Stacey, Vaithehy Shanmugam, Martina Sharman, Jacqui Troughton, Sian Williams. The STAND study is a MRC (NPRI) funded study (Project #91409). The funder has not had any role in data collection, analysis or manuscript preparation.

### Author details

[1]Diabetes Research, Department of Cardiovascular Sciences, University of Leicester, University Hospitals of Leicester NHS Trust, Leicester Royal Infirmary, Leicester LE1 5WW, UK. [2]Diabetes Research Department, University Hospitals of Leicester, Leicester, UK. [3]School of Sport, Exercise and Health Sciences, Loughborough University, Loughborough LE11 3TU, Leicestershire, UK. [4]Department of Health Sciences, University of Leicester, 22-28 Princess Road West, Leicester LE1 6TP, UK.

### Authors' contributions

The original study proposal ideas were led by SJHB, MD, TG, KK, MN, and TY. EW and CE participated in the design, management and coordination of the study and drafted the manuscript. All have been involved in revising the content of the manuscript. All authors have read and approved the final manuscript.

### Competing interests

The authors declare that they have no competing interests.

Received: 31 October 2011 Accepted: 8 December 2011
Published: 8 December 2011

### References

1. Hsia Y, Neubert AC, Rani F, *et al*: An increase in the prevalence of type 1 and 2 diabetes in children and adolescents: results from prescription data from a UK general practice database. *Br J Clin Pharmacol* 2009, 67(2):242-249.
2. Haines L, Wan KC, Lynn R, Barrett TG, Shield JP: Rising incidence of type 2 diabetes in children in the UK. *Diab Care* 2007, 30(5):1097-101.
3. Feltbower RG, McKinney PA, Campbell FM, Stephenson CR, Bodansky HJ: Type 2 and other forms of diabetes in 0-30 year olds: a hospital based study in Leeds, UK. *Arch Disease Child* 2003, 88:676-679.
4. Millett C, Khunti K, Gray J, Saxena S, Netuveli G, Majeed A: Obesity and intermediate clinical outcomes in diabetes: evidence of a differential relationship across ethnic groups. *Diab Med* 2008, 25:685-691.
5. McGavock J, Sellers E, Dean H: Physical activity for the prevention and management of youth-onset type 2 diabetes mellitus: focus on cardiovascular complications. *Diab Vasc Dis Res* 2007, 4(4):305-310.
6. Hillier TA, Pedula KL: Complications in young adults with early-onset Type 2 diabetes. *Diab Care* 2003, 26:2999-3005.
7. Owen N, Healy GN, Matthews CE, Dunstan DW: Too much sitting: the population health science of sedentary behavior. *Exer Sport Sci Rev* 2010, 38(3):105-113.
8. Pate RR, O'Neill JR, Lobelo F: The evolving definition of "sedentary". *Exer Sport Sci Rev* 2008, 36(4):173-178.
9. Healy GN, Matthews CE, Dunstan DW, Winkler EAH, Owen N: Sedentary time and cardio-metabolic biomarkers in US adults: NHANES 2003-06. *Eur Heart J* 2011, 32(5):590-597.
10. Morris JN, Heady JA, Raffle PA, Roberts CG, Parks JW: Coronary heart-disease and physical activity of work. *Lancet* 1953, 265(6796):1111.
11. Hamilton MT, Hamilton DG, Zderic TW: Role of low energy expenditure and sitting in obesity, metabolic syndrome, Type 2 diabetes, and cardiovascular disease. *Diabetes* 2007, 56:2655-2667.
12. Dunstan DW, Barr ELM, Healy GN, Salmon J, Shaw JE, Balkau B, Magliano DJ, Cameron AJ, Zimmet PZ, Owen N: Television viewing time and mortality: the Australian Diabetes, Obesity and Lifestyle Study (AusDiab). *Circulation* 2010, 121:384-391.
13. Dunstan DW, Salmon J, Owen N, Armstrong T, Zimmet PZ, Welborn TA, Cameron AJ, Dwyer T, Jolley D, Shaw JE: Physical activity and television viewing in relation to risk of undiagnosed abnormal glucose metabolism in adults. *Diab Care* 2004, 27:2603-2609.
14. Katzmarzyk PT, Church TS, Craig CL, Bouchard C: Sitting time and mortality from all causes, cardiovascular disease, and cancer. *Med Sci Sport Exer* 2009, 41(5):998-1005.
15. Davies MJ, Heller S, Skinner TC, Campbell MJ, Carey ME, Cradock S, *et al*: Effectiveness of the diabetes education and self management for ongoing and newly diagnosed (DESMOND) programme for people with newly diagnosed type 2 diabetes: cluster randomised controlled trial. *BMJ* 2008, 336(7642):491-495.
16. Yates T, Davies MJ, Gorely T, Bull F, Khunti : Rationale, design and baseline data from the prepare (prediabetes risk education and physical activity recommendation and encouragement) programme study: a randomized controlled trial. *Pat Ed Couns* 2008, 73:264-271.
17. National Institute for Clinical Excellence: *Guidance on the use of patient-education models for diabetes (Technology Appraisal Guidance 60)* London: Author; 2003 [http://www.nice.org.uk/nicemedia/live/11496/32610/32610.pdf].
18. Lawton J, Rankin D: How do structured education programmes work? An ethnographic investigation of the dose adjustment for normal eating (DAFNE) programme for type 1 diabetes patients in the UK. *Soc Sci Med* 2010, 71(3):486-493.
19. Bartholomew LK, Parcel GS, Kok G, Gottlieb NH: *Planning health promotion programs: An intervention mapping approach.* 2 edition. San Francisco, CA: John Wiley; 2006.
20. Craig P, Dieppe P, Macintyre S, Michie S, Nazareth I, Petticrew M: Developing and evaluating complex interventions: the new medical research council guidance. *BMJ* 2008, 337:a1655.
21. Leventhal H, Meyer D, Nerenz D: The common-sense representation of illness danger. In *Contributions to Medical Psychology.* Edited by: Rachman S. New York: Pergamon; 1980:7-30.
22. Bandura A: *Social foundations of thought and action: A social cognitive theory* Englewood Cliffs, NJ: Prentice Hall; 1986.

23. Yates T, Davies M, Gorely T, Bull F, Khunti K: **Effectiveness of a pragmatic education program designed to promote walking activity in individuals with impaired glucose tolerance.** *Diab Care* 2009, **32(8)**:1404-1410.
24. Gollwitzer PM: **Implementation intentions: strong effects of simple plans: how can good intentions become effective behavior change strategies?** *Am Psychol* 1999, **54**:493-503.
25. Epstein LH, Roemmich JN: **Reducing sedentary behaviour: role in modifying physical activity.** *Exer Sport Sci Rev* 2001, **29(3)**:103-108.
26. Skinner T, Davies M, Heller S, Khunti K: **To determine the effects of a structured educational programme on illness beliefs, quality of life and physical activity in individuals with newly diagnosed type 2 diabetes: results from the DESMOND (Diabetes education and self-management for ongoing and newly diagnosed) pilot study.** *Diab Med* 2005, **22(Suppl 2)**:15.
27. Healy GN, Wijndaele K, Dunstan DW, Shaw JE, Salmon J, Zimmet PZ, *et al*: **Objectively measured sedentary time, physical activity, and metabolic risk: the Australian Diabetes, Obesity and Lifestyle Study (AusDiab).** *Diab Care* 2008, **31**:369-371.
28. Plasqui G, Westerterp KR: **Physical activity assessment with accelerometers: an evaluation against doubly labeled water.** *Obesity* 2007, **15**:2371-2379.
29. Healy GN, Clark BK, Winkler EAH, Gardiner PA, Brown WJ, Matthews CE: **Measurement of adults' sedentary time in population-based studies.** *Am J Prev Med* 2011, **41(2)**:216-227.
30. Matthews CE, Chen KY, Freedson PS, Buchowski MS, Beech BM, Pate RR, Troiano RP: **Amount of time spent in sedentary behaviors in the United States, 2003-2004.** *Am J Epid* 2008, **167**:875-881.
31. Rosenberg DE, Bull FC, Marshall AL, Sallis JF, Bauman AE: **Assessment of sedentary behavior with the international physical activity questionnaire.** *J Phys Act Health* 2008, **5(Suppl 1)**:S30-S44.
32. Freedson PS, Melanson E, Sirard J: **Calibration of the computer science and applications, inc. accelerometer.** *Med Sci Sports Exerc* 1998, **30**:777-781.
33. Grant PM, Ryan CG, Tigbe WW, Granat MH: **The validation of a novel activity monitor in the measurement of posture and motion during everyday activities.** *Br J Sports Med* 2006, **40(12)**:992-997.
34. Kozey-Keadle S, Libertine A, Lyden K, Staudenmayer J, Freedson P: **Validation of wearable monitors for assessing sedentary behavior.** *Med Sci Sports Exerc* 2011, **43(8)**:1561-1567.
35. Hart TL, Ainsworth BE, Tudor-Locke C: **Objective and subjective measures of sedentary behavior and physical activity.** *Med Sci Sports Exerc* 2011, **43(3)**:449-456.
36. Craig CL, Marshall AL, Sjostrom M, Bauman AE, Booth ML, Ainsworth BE, Pratt M, Ekelund U, Yngve A, Sallis JF, Oja P: **International physical activity questionnaire: 12-country reliability and validity.** *Med Sci Sports Exerc* 2003, **35(8)**:1381-1395.
37. Friedewald W, Levy R, Fredrickson D: **Estimation of the concentration of low density lipoprotein cholesterol in plasma, without use of the preparative ultracentrifuge.** *Clin Chem* 1972, **18**:499-502.
38. Kind P, Dolan P, Gudex C, Williams A: **Variations in population health status: results from a United Kingdom national questionnaire survey.** *BMJ* 1998, **316**:736-741.
39. Broadbent E, Petrie KJ, Main J, Weinman J: **The brief illness perception questionnaire.** *J Psychosom Res* 2006, **60**:631-637.
40. Keller C, Fleury J, Gregor-Holt N, Thompson T: **Predictive ability of social cognitive theory in exercise research: an integrated literature review.** *Online J Know Synth Nursing* 1999, **6**:2.
41. Chalder T, Berelowitz G, Pawlikowska T, Watts L, Wessely S, Wright D, *et al*: **Development of a fatigue scale.** *J Psychosom Res* 1993, **37(2)**:147-153.
42. Buysse DJ, Reynolds CF, Monk TH, Berman SR, Kupfer DJ: **The Pittsburgh sleep quality index: a new instrument for psychiatric practice and research.** *Psychiat Res* 1989, **28(2)**:193-213.
43. Zigmond AS, Snaith RP: **The hospital anxiety and depression scale.** *Acta Psychiatr Scand* 2006, **67**:361-370.

## Pre-publication history

The pre-publication history for this paper can be accessed here:
http://www.biomedcentral.com/1471-2458/11/908/prepub

doi:10.1186/1471-2458-11-908
**Cite this article as:** Wilmot *et al.*: Rationale and study design for a randomised controlled trial to reduce sedentary time in adults at risk of type 2 diabetes mellitus: project STAND (*Sedentary Time ANd Diabetes*). *BMC Public Health* 2011 **11**:908.

**Submit your next manuscript to BioMed Central and take full advantage of:**

- **Convenient online submission**
- **Thorough peer review**
- **No space constraints or color figure charges**
- **Immediate publication on acceptance**
- **Inclusion in PubMed, CAS, Scopus and Google Scholar**
- **Research which is freely available for redistribution**

Submit your manuscript at
www.biomedcentral.com/submit

BioMed Central
