# Supplementary material for: A Randomised Controlled Trial to Reduce Sedentary Time in Young Adults at Risk of Type 2 Diabetes Mellitus: Project STAND (Sedentary Time ANd Diabetes)
Source: PLoS One. 2015 Dec 1;10(12):e0143398. doi: 10.1371/journal.pone.0143398 (PMC4666612; doi:10.1371/journal.pone.0143398)
Supplement: S1 Protocol — (DOC) [file pone.0143398.s003.doc]

**
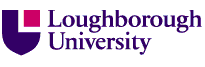

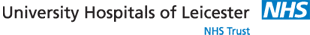

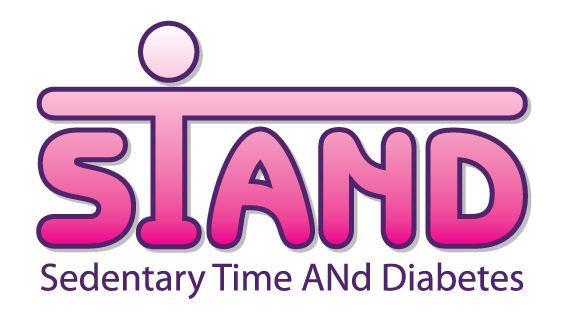

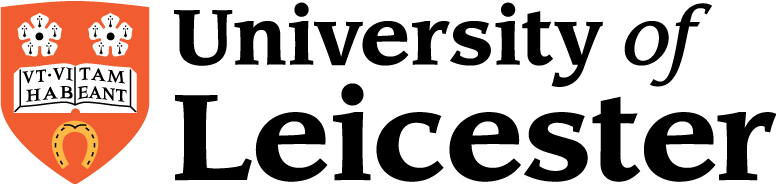
**

**Project STAND**

**Protocol**

An Intervention to Decrease Sedentary Behaviour in Young

Adults at Risk of Type 2 Diabetes Mellitus: Project STAND (Sedentary Time ANd Diabetes)

**Chief Investigator:**

Prof Stuart Biddle

Loughborough University

**UHL Principal Investigator:**

Prof Melanie Davies

University of Leicester

**Study Co-ordinators:**

Dr Emma Wilmot

Dr Charlotte Edwardson

**Co-Investigators:**

Dr Marian Carey

Dr Trish Gorely

Prof Kamlesh Khunti

Prof Myra Nimmo

Ms Jacqui Troughton

Dr Tom Yates

**Trial Sponsor:**

University Hospitals of Leicester (UHL)

#### ABSTRACT

**Relevance to diabetes:** The rising prevalence of obesity and sedentary behaviour has lead to an epidemic of Type 2 Diabetes (T2DM). As the health and economic costs of T2DM continue to increase, there is an urgent need for effective lifestyle interventions to prevent the development of T2DM. Our group has previously developed successful structured education programmes including DESMOND which improved lifestyle, depression, illness beliefs, weight and cardiovascular risk in adults with T2DM and the PREPARE programme which resulted in increased activity levels and reduced 2 hour glucose values in people with prediabetes. Our proposed study will assess the effect of theory driven structured education, facilitated using automated technology, on sedentary behaviour and health outcomes in young adults at risk of T2DM.

**Methods:** Inclusion criteria: Age 18-40 years with a BMI ≥30 (27.5 for South Asians) *or* age 18-40 years with a BMI≥25kg/m^2^ (>23kg/m^2^ for South Asians) plus one additional risk factor: first degree family history of diabetes or cardiovascular disease; previous gestational diabetes; polycystic ovarian syndrome; HbA1c ≥5.8% or impaired glucose regulation (defined as impaired glucose tolerance or impaired fasting glucose). Participants will be randomised to a control (C) or intervention (I) group, the latter given structured education, based on the PREPARE and DESMOND programmes, with the aim of reducing sedentary behaviour. The structured education programme will incorporate movement technology to facilitate participant feedback and self-monitoring**.**

**Outcomes:** The primary outcome of the intervention trial will be reduced sedentary behaviour, measured by an accelerometer at 12 months. Secondary outcome measures will include physical activity, sitting/lying time, oral glucose tolerance tests, lipoprotein lipase, inflammatory biomarkers, body weight, waist circumference, blood pressure, illness perceptions and efficacy beliefs for behaviour change.

**Expected outcome:** This will be the first UK trial to address sedentary behaviour change in a population of younger adults at risk of T2DM. Our results will provide a platform for the development of a range of future multidisciplinary interventions in this rapidly expanding high-risk population.

**BACKGROUND AND RATIONALE**

National Service Frameworks Standards 1 and 2 emphasise the importance of prevention and early detection of Type 2 Diabetes Mellitus (T2DM). However, little progress has been made in these areas, in contrast to that of the clinical care of established cases. As a result of increasing obesity and high levels of sedentary behaviour, there has been a recent explosion in the prevalence of T2DM in the younger age groups. There is an urgent need for effective lifestyle interventions to prevent progression to diabetes in young, at risk, adults. There is mounting evidence that sedentary behaviour is associated with both diabetes and obesity. This programme of work will determine the effectiveness of a structured education and lifestyle intervention, aimed at reducing sedentary behaviour, in young adults at risk of T2DM.

**TYPE 2 DIABETES IN THE YOUNG**

The epidemic of obesity in western populations has led to a dramatic increase in the incidence of T2DM. Until recently T2DM was considered a disease of older adults. We now see the condition in children and young adults (Ehtisham, 2000, Ehtisham 2004, Haines 2007, Kirk 2000). While type 1 remains the main form of diabetes in young people, it is anticipated that T2DM will be the predominant form within the next 10 years in some ethnic groups (Alberti 2004). The young person with T2DM often has specific issues, representing an extreme phenotype. They are likely to be obese, have a strong family history of T2DM, lead a sedentary lifestyle, be of black or minority ethnic (BME) origin and come from less affluent socio-economic groups (Haines 2007, Feltbower 2003, Millett 2008). For the individual, it heralds a marked risk of progression to a range of micro and macrovascular complications (McGavock 2007). At the time of diagnosis, 7-22% of young people with T2DM have microalbuminuria, 82% have abnormal lipids and 30-55% have hypertension (McGavock 2007). The hazard of developing a myocardial infarct in early-onset T2DM (<45 years) is 4-fold higher than in late onset T2DM (>45 years) and 14-fold higher than in people without diabetes (Hillier 2003). From our diabetes specialist clinic at one site in UHL we have over 30 cases of T2DM in those up to 25 years and 185 in those aged 35 or younger (data from UHL Clinical Workstation March 2009). From a societal perspective, the explosion of young people developing T2DM has significant implications for future workforce and health care systems. It is crucial, therefore, that effective lifestyle interventions are developed and employed to prevent the development of diabetes in young adult at-risk groups.

**PHYSICAL ACTIVITY AND SEDENTARY BEHAVIOUR**

Physical activity (PA) and sedentary behaviour are recognised as important lifestyle factors affecting health and well being (Khunti 2007, 2008, Stone 2007, Stone 1998). Sedentary behaviour – essentially ‘sitting time’ - occupies a very large part of the day. Even if individuals meet the national guidelines for PA by being at least moderately active for 30 minutes per day throughout the week, it is highly likely that for the other 23.5h they will be predominantly sedentary. Recent findings suggest that sedentary behaviour may be an important precursor of chronic disease in its own right, independent of PA (Ekelund 2006). For example, three large UK data sets show that TV viewing (the most prevalent sedentary behaviour), independent of PA, is associated with BMI, obesity or other metabolic markers, either cross-sectionally (Jakes 2003) or prospectively (Parsons 2008, Viner 2005). Similar findings have been seen in Australia (Dunstan 2007, Healy 2008, Sugiyama 2008), USA (Ford 2005, Hu 2003, Eisenmann 2008), Europe (Wijndaele 2009), and Taiwan (Chang 2008), showing the importance of studying sedentary behaviour in its own right. Sedentary ‘opportunities’ in our society are ubiquitous as new technologies and attractive home-based entertainment become more widely available. Moreover, many occupations involve large amounts of time sitting. Therefore, understanding how to reduce sedentary behaviour could have significant public health benefits.

**SEDENTARY BEHAVIOUR AND INFLAMMATION**

Of novel interest is the link between inactivity and systemic chronic low grade inflammation. As with other physical activity interventions, the focus of research has been on the introduction of exercise regimens to offset the associated cardiovascular risk (Yates 2008) and to our knowledge there are no experimental studies investigating the effects of a reduction of sitting time on these systems. T2DM and CVD are characterized by chronic low-grade inflammation (Pickup 1998). Circulating levels of adipocytokines are predominantly influenced by levels of adiposity and have been proposed as an important meditating link between obesity and chronic disease (Berg 2005). The elevations are believed to be related to the accumulation of macrophages in adipose tissue (Weisberg 2003) and to a distortion of the transition from innate to acquired immunity (Jones 2005). A key regulator in this switch is the inflammatory cytokine IL6 (Hoebe 2004), which is also released in large quantities on exposure to exercise (Febbraio 2002). The biological activity of IL6 is dependent on it combining with the receptor IL6R. Where tissues are devoid, or have a low level of activity of this receptor, IL6 can combine with the soluble form sIL6R and act on tissues through a process termed trans-signalling. IL6R and sIL6R have been shown to increase with acute activity (Keller 2005; Gray et al 2009) although with chronic exercise IL6R increases (Keller 2005) whilst sIL-6R decreases (Silverman 2009) and have a significant role to play in the aetiology of chronic disease, and in particular in T2DM (Qi 2009). Understanding the effect of reductions in sedentary behaviour on IL6R and sIL6R could therefore make a significant contribution to knowledge in this field.

**SEDENTARY BEHAVIOUR: THE MECHANISM**

Hamilton and colleagues (Hamilton 2007) argue the case for a greater emphasis on ‘inactivity physiology’, in contrast to the more usual ‘exercise physiology’. This is supported by a series of papers addressing the activity of lipoprotein lipase (LPL) in a rat hind limb model in response to different activity stimuli. Animal models demonstrate that after 12 hours of decreased activity, there is a partial reduction in lipoprotein lipase (LPL) function. LPL has a central role in lipid metabolism and evidence has shown that a partial reduction in LPL function is associated with a 5-fold increase in the odds for death and CHD (Wittrup1999). A rat model study shows a reduction in LPL after 4h of hind limb unloading with a high density lipoprotein cholesterol concentration and a local reduction of plasma triacylglycerol after 24h (Bey 2003). These biochemical studies lend support to behavioural studies that would indicate a link between cardiovascular risk factors and prolonged sitting (Hamilton 2008). To date, there have been no human intervention human studies to assess the impact of reducing sedentary time on lipid fractions, LPL or other metabolic risk factors.

**STRUCTURED EDUCATION**

Structured self-management education is recognised as the cornerstone of effective lifestyle self-management. Our group has previously developed structured education programmes which have demonstrated benefit in randomised controlled trials. DESMOND - Diabetes Education and Self Management for Ongoing and Newly Diagnosed is a diabetes group structured education programme designed for adults with recently diagnosed T2DM. DESMOND successfully improved lifestyle, depression, illness beliefs, weight and modelled CV risk in a 12 month randomised controlled trial (Davies 2008). As such DESMOND has now been rolled out nationally and is delivered in over 70 primary care organisations throughout the UK. PREPARE - Pre-diabetes Risk Education and Physical Activity Recommendation and Encouragement, another group structured education course designed and tested by our group in Leicester, was designed to increase walking activity in adults with prediabetes. The PREPARE programme comprised a group-based, person centred, education programme which targeted perceptions, knowledge, self-efficacy, barriers, and self-regulation. In a randomised controlled trial, the PREPARE programme successfully increased physical activity levels and reduced 2 hour glucose values in adults with prediabetes at 3 and 12 months (Yates 2009). However, these existing education programmes were designed for the older adult and do not take into account the specific issues facing younger adults. There is an urgent need to develop effective lifestyle self management programmes for younger adults at risk of T2DM in the UK to reduce sedentary behaviour and its associated negative outcomes. The PREPARE programme will be modified in this programme of work to focus on reducing sedentary behaviour.

**INTERVENTION MAPPING**

Our group, in the development of the above programmes, have identified appropriate health behaviour theories (from a range of over 20) on which to base structured education programmes using the core processes proposed by Bartholomew’s intervention mapping protocol (Bartholomew 2001). Intervention mapping is a systematic approach to developing health education programmes that provide a useful and coherent method for identifying which theoretical determinants are likely to be important in the development of an intervention (Bartholomew 2001). This approach, which will be used to develop the Sedentary Time and Diabetes (STAND) intervention, ensures that empirical evidence is used to confirm or reject a broad range of potentially useful theoretical domains that are not necessarily confined to a particular theory or theories, thus ensuring identification of a comprehensive set of domains that are likely to be important in the promotion of a given health behaviour.

Core steps of the Intervention Mapping process are:

1. Conduct a needs assessment
2. Create matrices of change objectives based on the determinants of behaviour and environmental conditions
3. Select theory-based intervention methods and practical strategies
4. Translate methods and strategies into an organised programme
5. Plan for adoption, implementation and sustainability of the programme
6. Generate an evaluation plan

During the development of the PREPARE programmes (Yates 2008) our group identified key psychological theories which underpin successful physical activity and multi-factor intervention programmes in individuals with and at risk of diabetes.. These theories include:

- - Bandura’s (1986) Social Cognitive Theory, including self-efficacy and self-regulation
  - Gollwitzer’s (1999) implementation intentions - identified as an important framework for developing successful strategies around self-regulation and translating intentions into behaviour.
  - Leventhal’s (1980) Common Sense Model - thought to be relevant and instructive for targeting risk perceptions.
  - Chaiken’s (1987) Dual Process Theory

Working in parallel, the DESMOND group identified the same key theories, which are viewed as a crucial element of these successful interventions These theories which have been integrated into a single theoretical model of behaviour change in the DESMOND and PREPARE programmes will be utilised in the development of the STAND intervention.

**COMPLEX INTERVENTIONS**

The STAND structured education programme is aimed at reducing sedentary behaviour in a multi-ethnic population of young adults at risk of diabetes. This programme will be developed in line with the Medical Research Council’s framework for developing and evaluating complex interventions to improve health (Craig 2008). This framework describes how evidence, theory, modelling, and exploratory trials should be used iteratively to develop complex interventions. Importantly this framework also states that complex interventions sometimes need to be adapted to local circumstances rather than being completely standardized; therefore, we will tailor the STAND structured education programme to the needs of the young adult multi-ethnic community of the UK.

This will be the first UK trial to address sedentary behaviour change in a population of young adults at risk of T2DM, and possibly the first in the world. It is a proof-of-concept trial and, if successful, will be developed into a more complex trial to allow for the testing of other behaviours alongside sedentary behaviour, such as physical activity and dietary modification. The trial will inform behaviour change programmes for this at-risk group, and provide a major new direction of behaviour change, complementing the more conventional approach of encouraging increases in moderate-to-vigorous physical activity. In addition, if shown to be successful, the study will inform the focus of advice and programmes delivered in schools for people about to embark on early adulthood and employment. The results will have implications for the approaches taken in worksite settings concerning the neglected area of strategies employed to reduce sedentary behaviour (for example, for breaking up sedentary time; office design and work patterns).

**METHODOLOGY**

Using the MRC framework for complex interventions our proposed programme of work will comprise three phases:

**PHASE 1:** Qualitative data collection and systematic review

**PHASE 2:** Development and pilot of an evidence based structured self-management education programme, combining phase 1 data with robust psychological theory.

**PHASE 3:** Randomised controlled multicentre education and lifestyle intervention trial.

**PHASE 1: COLLECTION OF QUALITATIVE DATA**

**AIMS**

Using qualitative methods (interviews and focus groups) we aim to characterise the health beliefs and needs of young adults at risk of T2DM. The qualitative data and psychological theories which form the basis of previously successful education programmes (DESMOND, PREPARE) will provide a platform for the development of a tailored self-management structured education course for young adults at risk of T2DM. The purpose of the qualitative data collection is to:

- gain a detailed understanding of participants’ views and perceptions of diabetes risk
- assess their perception of the acceptability and efficacy of a lifestyle intervention for the reduction in sedentary behaviour

**METHODS**

*INTERVIEWS AND FOCUS GROUPS:*

The choice between gaining feedback through an interview or focus group will be negotiated with the participants and the option of a telephone interview will also be available. Interviews and group discussions will be audio recorded and transcribed. Topic guides will be used to facilitate the process of qualitative data collection, but these will be used flexibly, with scope for discussion of additional relevant topics that may arise and revision of the topic guide in line with any additional emergent issues. The initial schedules will be developed as part of the study based on feedback from pilot qualitative interviews and focus groups, but it is anticipated that data collection from patients will include discussion of topics such as sedentary behaviour, physical activity and associated barriers, perception of risk, healthy eating and novel behaviour change technologies such as MiLife.

*GAINING FEEDBACK ON* *NOVEL BEHAVIOUR CHANGE TECHNOLOGIES:*

A novel method in the intervention is the use of technology to allow participants to self-regulate their sedentary behaviour. Participants will be asked to use behaviour change technology in their own home for a period of time. We will then use qualitative research methods (focus groups, interviews) to assess the usefulness of this as a tool in the STAND intervention. We will trial a range of suitable internet based behaviour change technologies and based on participant feedback, will select the most suitable device for the study.

One example of the possibilities we will investigate is Milife (www.milife.com). MiLife is an evidence-based automated internet based behaviour change system which has been shown to increase physical activity in adults (Hurling 2007). It combines behavioural psychology, artificial intelligence, advanced diagnostics, evidence-based weight management techniques and user-centred design to promote active lifestyles. MiLife can serve as a motivational tool for behaviour change. The technology involves the wearing of a wristwatch that accommodates a Bluetooth accelerometer and web based facilities.

A further possibility is the PAM coach (www.pam.com). This is a waist worn accelerometer which allows participants to monitor their activity in the ‘living’, ‘health’ and ‘sport’ zone. Their activity is converted into PAM points and they are able to set weekly PAM activity goals. The advantage of the PAM coach over MiLife is that it collects data from time spent in the “light” activity range which will allow participants to more accurately record any reduction in their sedentary behaviour.

In Phase 1, both technologies will be tried and tested within the target population. The overall aim is that the technology will be used for prompting, goal setting and self-monitoring to boost the effect of the workshops in the randomised controlled trial.

**SAMPLE SIZE AND RECRUITMENT**

Our final intervention will be delivered to a diverse group of people from a range of age groups and ethnicities. It is therefore important that these user groups are involved in the development of our intervention. We will ensure that focus groups and interviews are from a heterogeneous sample, with people from a representative range of age and ethnicities. Qualitative data will be collected until the point of data saturation where no new themes are emerging. Although it is difficult to estimate the point at which data saturation will occur, we anticipate that up to 40 participants and their partners and family members will need to be recruited to inform the development of our intervention. Participants will be recruited via the Primary Care and Diabetes local research networks.

**INCLUSION CRITERIA**

Age 18-40 years plus a BMI ≥30kg/m^2^ (≥27.5kg/m^2^ for south Asians)

***OR***

Age 18-40 years with a BMI ≥25kg/m^2^ (≥23kg/m^2^ for south Asians) plus one of:

- - family history of diabetes or cardiovascular disease in a first degree relative;
  - previous gestational diabetes;
  - polycystic ovarian syndrome;
  - HbA1c ≥5.8% (from our local Addition Leicester diabetes screening data a cut off HbA1c of 5.8% provided the best sensitivity and specificity for a diagnosis of prediabetes).
  - Impaired glucose regulation

Defined according to the World Health Organisation as impaired glucose tolerance (2 hour glucose test from 7.8mmol/l to 11mmol/l following standard oral glucose tolerance test) *or* impaired fasting glucose (glucose value from 6.1 to 6.9mmol/l)

**DATA ANALYSIS**

A process based on ‘Framework Methodology’ will be used to analyse the data collected, including familiarisation through reading and re-reading transcripts; development of a coding frame based on key themes that have been identified; detailed indexing (coding) of transcripts; charting to organise and summarise data; followed by a review of the charted data to facilitate interpretation. The main focus of the process of interpretation will be to inform the development of the educational intervention. However, we will also consider more broadly any emerging themes and patterns, for example, in relation to motivation and other facets of behaviour. The initial coding frame will be agreed after review of a selection of transcripts. A constant comparative approach will be adopted whereby the coding frame will be open to revision during the detailed coding and charting stages of the process of analysis. Data collection will end at the point of data saturation whereby new themes are no longer emerging and those identified have been fully explored.

**PHASE 2: DEVELOPMENT AND PILOT OF THE STAND INTERVENTION**

This is a proof-of-concept intervention and as such we plan to conduct extensive modelling and pilot work to comply with the MRC’s framework for complex interventions (Craig 2008). This will involve exploring appropriate methods of behaviour change and content of the workshops, in particular, and seeking the views of participants in establishing methods for behavioural prompting and regulation. Based on these data, we will design the intervention to be implemented.

**Figure 1**. MRC framework for complex interventions: Key elements of the development and evaluation process (Craig, 2008)


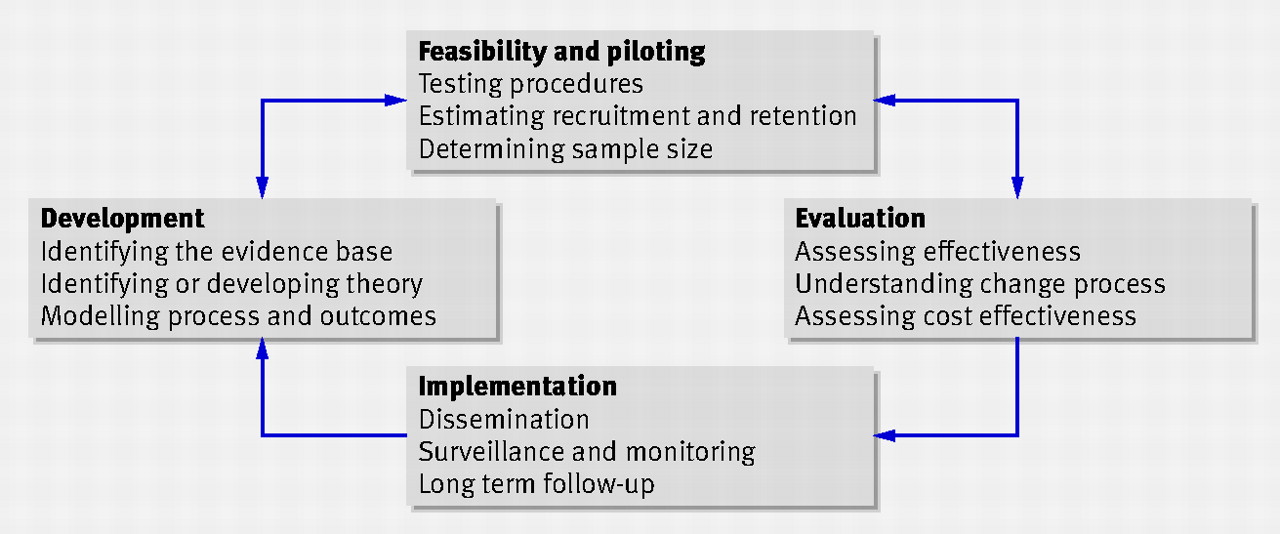


**INTERVENTION DESIGN**

The STAND intervention will be an education workshop aimed at behaviour change, and is based on the DESMOND and PREPARE programmes (Skinner 2006, Davies 2008, Yates 2008a, 2008b). Both these interventions have been extensively piloted and then delivered in randomised controlled trials. These programmes will be adapted to target a reduction in sedentary behaviour in young adults. Modifications will be overseen by investigators with expertise in health behaviour theory, and will take into account participant preferences gathered from qualitative work in Phase 1, consistent with MRC guidelines for complex interventions (Craig 2008).

The STAND group education programme will have a written curriculum suitable for the broadest range of participants. Registered healthcare professionals will receive formal training to deliver the programme and quality assurance will be evaluated via internal and external assessment to ensure consistency of delivery. Learning will be elicited rather than taught, with the behaviour of the educators promoting a non-didactic approach. The content of the curriculum will focus on lifestyle factors such as physical activity, sedentary behaviour, behaviour change technologies, food choices and cardiovascular risk factors. The detail of the course content, duration and location will be informed by the qualitative work in Phase 1.

Behaviour change will be encouraged through the use of self-regulatory strategies, for example by increasing the confidence of subjects to reduce sedentary behaviour, and by encouraging their awareness of how to make less sedentary options more attractive and available, as well as limiting time in some sedentary pursuits. This may seem rather simple, but it will be challenging to help people become aware of, and then adopt changes to, ingrained behaviours that address prolonged sedentary time and that are likely to be routine and habitual. Strategies for participants will be based on Social Cognitive Theory (Bandura 1986) and Behavioural Choice Theory (Epstein 2001) both having been used in sedentary behaviour change, but will also be informed by the ‘Common Sense Model’ of illness perceptions (Hagger 2003), as in PREPARE.

**EDUCATOR TRAINING & QUALITY ASSURANCE**

We will develop comprehensive educator training, quality assurance, and a development programme for Project STAND. This will be based on established protocols and infrastructure developed by the DESMOND collaborative [www.desmond-project.org.uk](http://www.desmond-project.org.uk/) (Davies 2008).

**PILOT STAND STRUCTURED EDUCATION COURSES**

Once developed, the course will be piloted with user groups and refined as required. Our group has previous expertise in the quality development of similar interventions (Skinner 2008, Yates 2008). Participants who meet the inclusion criteria outlined in Phase 1 will be recruited from primary care and the wider community. Participants will be recruited from the wider community using posters and media adverts. These will be distributed in supermarkets, Universities, hospitals etc. We will also approach large local companies within the Leicestershire region with a view to distributing posters and emails alerting their staff of the opportunity to participate in the study. They will attend a pilot STAND structured education course. At the end of the course information on the useful and less useful aspects of the course will be gathered from the participants (via questionnaires and interviews). This information will be used to refine the content and delivery of the course. Further pilot courses will be run until the research team are satisfied that the structured education course is fit for purpose.

**PHASE 3: RANDOMISED CONTROLLED TRIAL**

In Phase 3, the STAND group structured education course will be delivered in a randomised controlled trial to 178 participants from across Leicestershire and the South East Midlands Diabetes research network.

**HYPOTHESIS**

The primary hypothesis of the STAND randomised controlled trial is:

- Structured education can decrease sedentary behaviour in young adults at risk of T2DM

The secondary hypothesis is:

- Reduced sedentary behaviour results in favourable changes in key behavioural and biological markers of T2DM risk

**INCLUSION CRITERIA**

Participants who meet the following inclusion criteria will be recruited from primary care and the wider community:

Age 18-40 years plus a BMI ≥30kg/m^2^ (≥27.5kg/m^2^ for south Asians)

OR

Age 18-40 years with a BMI ≥25kg/m^2^ (≥23kg/m^2^ for south Asians) plus one of:

- - family history of diabetes or cardiovascular disease in a first degree relative;
  - previous gestational diabetes;
  - polycystic ovarian syndrome;
  - HbA1c ≥5.8% (from our local Addition Leicester diabetes screening data a cut off HbA1c of 5.8% provided the best sensitivity and specificity for a diagnosis of prediabetes).
  - Impaired glucose regulation

Defined according to the World Health Organisation as impaired glucose tolerance (2 hour glucose test from 7.8mmol/l to 11mmol/l following standard oral glucose tolerance test) *or* impaired fasting glucose (glucose value from 6.1 to 6.9mmol/l)

**EXCLUSION CRITERIA**

Due to the nature of the intervention, our exclusion criteria are as follows:

1. Physical condition which limits full participation in the study;
2. Active psychotic illness or a significant illness which, in the view of the investigators, would prevent full participation;
3. Inability to communicate in verbal and written English;
4. Steroid use;
5. Diabetes;
6. Pregnancy.

**RECRUITMENT: PRIMARY CARE**

Our initial strategy will be recruitment of participants from primary care, co-ordinated via the Primary Care Research Network (East Midlands and South Yorkshire (PCRN EMSY). Our group have established strong links with primary care, having recruited 8579 patients for ADDITION (Sandbaek 2008) in Leicester alone. We will use these existing links to recruit participants meeting the inclusion criteria (identified from GP data bases) from primary health care practices that reflect the diverse ethnic and socio-economic makeup of the Leicester, Northampton and Kettering regions. While we recognize that BMI is not routinely collected in some parts of the UK, there is a Locally Enhanced Service (LES) in place in Leicester City PCT that financially rewards individual practices for collecting BMI data, therefore BMI is now routinely captured in many primary health care practices within the region. We will recruit from up to 40 primary health care practices with an expected average list size of 6000 patients. Based on local practice records and census data we expect that about 10% of each practice population will meet our age range criteria, of which around 8-15% will meet the additional inclusion criteria given the high prevalence of diabetes and cardiovascular disease within the Leicester region. Based on previous research, we would expect that 20-30% of eligible individuals will consent to take part in the study. Practices will be recruited on an ongoing basis until the final sample size is reached. Around 30% of participants will be from a south Asian ethnic background, and 50-60% will be male.

Eligible individuals will be contacted by a member of their primary health care practice on behalf of our research team and invited to take part in the study. On agreement to take part they will be assigned a study number and identifiable data will be stored securely.

We will also contact Leicester ADDITION study participants who meet the inclusion criteria. Invitation letters will be sent to the participants. If interested, potential participants will return a reply slip to the research team. Research staff will send out the participant information sheet. Initial written contact will be followed by a telephone call to discuss participation and the details of the first study appointment.

**RECRUITMENT: SECONDARY CARE**

One of the study inclusion criteria is a family history of diabetes. As such we will send study information to patients with diabetes who are currently attending UHL diabetes services, to ask them to pass the study patient information sheet to relatives aged 18-40 years who may be interested in taking part in the study. We will also search UHL clinical workstation to identify patients attending the diabetes and endocrinology department who meet the inclusion criteria. Invite letters will be sent to patients who meet the inclusion criteria.

**RECRUITMENT: COMMUNITY**

Participants will be recruited from the wider community using posters and media adverts. These will be distributed in supermarkets, Universities, hospitals etc. We will also approach large local companies within the East Midlands region (Leicestershire, Nottinghamshire, Derbyshire etc) with a view to distributing posters and emails alerting their staff of the opportunity to participate in the study. Potential participants will contact the research team directly. We will send them the participant information letter and they will have at least a week to decide whether they wish to participate. If they are agreeable, an appointment will be made for the first study visit.

All study participants will be fully reimbursed for their travelling expenses. In addition, we recognise that our target group are a hard to reach group with many pressures on their time (work, family etc). Therefore, we will provide participants with £20 for each study visit (1/2 day per visit) as a token of appreciation of their time.

**RANDOMISATION**

Once baseline data have been collected, participants will be randomised to either a control (C) or intervention (I) group, the latter being given the STAND structured education programme aimed at decreasing sedentary behaviour. Randomisation (stratified by age, sex, and ethnicity) will be set up by a statistician.

**INTERVENTION ARM**

Each individual in the intervention arm will participate in the tailored, group, STAND structured self-management education programme which will be developed in Phase 2. The intervention programmes will be delivered by trained educators. It is envisaged that the courses will be delivered in a community setting, at a location considered convenient for the participants. Although the content has yet to be developed, Table 1 provides an example of how the STAND structured education course may look (based on the outline of the PREPARE programme). The detail of each module and the time dedicated to each will be informed by the work done in Phases 1 and 2. At the end of the education course the participant will be shown how to use the behaviour change technology which will provide ongoing personalised feedback on their sedentary time.

At 6 weeks, intervention arm participants will be contacted by study staff via telephone to review their progress to support behavioural change maintenance.

**CONTROL ARM**

The control group will receive an information leaflet focusing on key illness perceptions of being at risk of T2DM, developed in the context of Leventhal’s common sense model. The leaflet will also focus on the importance of increasing physical activity and decreasing sedentary behaviour.

**Table 1:** Potential outline of the STAND structured education course which will be delivered to the participants in the intervention arm.

| **Module name** | **Main aims and educator activities** | **Theoretical underpinning** | **Time weighting** |
| --- | --- | --- | --- |
| ***Patient story*** | Give participants a chance to share their knowledge  and perception of sedentary behaviour in relation to diabetes risk and highlight any concerns  they may want addressed in the programme | Common sense model | 10%  (20 min) |
| ***Professional story*** | Use simple non-technical language, analogies, visual  aids and open questions to provide participants with  an overview of healthy glucose metabolism, the  aetiology of diabetes and the risk factors and  complications associated with diabetes  Help participants calculate their own individual  Diabetes risk scores | Common sense model  Dual process theory | 35%  (60 min) |
| ***Diet*** | Give participants an accurate understanding of the  link between diet and metabolic dysfunction | Social cognitive theory  Dual process theory | 15%  (25 min) |
| ***Sedentary behaviour*** | Use simple non-technical language, analogies,  visual aids and open questions to help participants:  identify how reducing sedentary behaviour may improve glucose control and body weight;  understand the current activity recommendations;  explore options for reducing sedentary behaviours in  everyday life; identify barriers to reducing sedentary behaviour; form action  plans and set personal goals  Practical demonstration of how to use the novel exercise technology for the self- regulation of sedentary time. | Social cognitive theory  Behavioural Choice Theory | 40%  (75 min) |

**OUTCOME MEASURES**

All primary and secondary outcome measures will be recorded at 0, 3 and 12 months.

**Primary Outcome:**

The primary outcome is a reduction in sedentary behaviour at 12 months. Sedentary behaviour will be objectively measured using the triaxial Actigraph GT3X model of accelerometer. These accelerometers are the most extensively validated and accurate on the market and they are the only commercially available accelerometers to correlate with energy expenditure as measured by double-labelled water (Plasqui, 2007).

Participants will be asked to wear the accelerometer on a waistband (in the right anterior axillary line) for ten consecutive days during waking hours. A total of 4 days valid wear will be required to count as a valid recording and a ‘valid day’ will consist of at least 10 hours of accelerometer movement data. The accelerometer will record movement data every 5 seconds (i.e., 5 second ‘epoch’). Prolonged periods of no movement, that is, strings of ‘0’, will be assumed to be non-wear time and will be excluded. The primary outcome measure is sedentary time defined as <100 counts per minute (Freedson 1998).

To encourage participants to remember to wear the accelerometer they will be provided with cards to place in convenient locations around the home. We will also send them text messages during data collection periods, reminding them to wear the device.

**Secondary outcomes:**

**Physical activity and body position**

- Accelerometer data
- Inclinometer data
- ActivPAL
- IPAQ questionnaire

Physical activity will be objectively measured using the accelerometer as described above. Secondary outcomes include steps per day, total body movement (counts per day), and time in light-, moderate- and vigorous-intensity physical activity as determined by counts per minute cut points proposed by Freedson et al (1998).

The Actigraph GT3X model incorporates an inclinometer which provides postural data. This has the advantage of allowing the research team to determine subject position (lying or sitting) and periods when the device has been removed.

The ActivPAL is a thigh worn accelerometer which measures the angle of the thigh, providing valuable data on participant posture (ie sitting or lying vs standing). The data obtained from this device will provide invaluable information about the time spent sitting. The ActivPAL will be worn on the thigh for the same 10 day period as the GT3X accelerometer.

The short ‘last-seven-days’ self-administered format of the International Physical Activity Questionnaire (IPAQ) will be used as a self-report measure of activity. This questionnaire provides a comprehensive measure of walking and other moderate- to vigorous-intensity activities carried out for more than 10 continuous minutes at work, in the home, as transport and during leisure time. IPAQ has been shown to have reasonable validity compared to accelerometer data (ρ ~ 0.4) and test-retest reliability (ρ ~ 0.7) in the UK when used as a measure of total moderate- to vigorous-intensity physical activity (Craig 2003). For this study, IPAQ will be used to measure total activity accumulated at work, for transport and in leisure time as well as an overall measure of moderate- to vigorous-intensity physical activity accumulated over all contexts.

### Biochemical variables

- Oral glucose tolerance test
- Blood U&E, lipid profile, liver and thyroid function tests, vitamin D
- Insulin, c-peptide, HbA1c, HOMA-B, HOMA-IR
- hsCRP, TNF alpha, sIL-6, and sIL-6R
- Biomarkers
- Genetics blood sample

Participants will be invited to attend each clinical measurement session after a 12-hour fast and 24 hours of avoiding vigorous intensity exercise.

This study will measure glucose control and insulin sensitivity using: fasting glucose, 2-hour post challenge glucose, fasting insulin, and HbA1c. In addition fasting lipid profile, highly sensitive C-reactive protein, key adipokines (interleukin 6 and its receptors, tumour necrosis factor alpha), urea & electrolytes (sodium, potassium, urea, creatinine) will be measured by venous sampling. This study includes markers of chronic inflammation and adipokines because these variables have been hypothesised to be directly involved in the pathogenesis of type 2 diabetes (Tataranni 2005) and have been shown to be inversely associated with overall physical activity and walking activity (Panagiotakos 2005, Yates 2008). However data on the relationship of these variables with sedentary behaviour is lacking. Therefore this study will further our understanding of the effect of sedentary behaviour change on markers of chronic low-grade inflammation.

In concordance with WHO recommendations those who have a fasting or 2-hour blood glucose level in the diabetes range at any clinical measurement session will be called back for a confirmatory oral glucose tolerance test. If diabetes is confirmed, the participant will be removed from the study and referred to their general practitioner for routine diabetes care.

All venepuncture and OGTT timings will be undertaken by trained phlebotomists who are not part of the scientific advisory team for this study and who are blinded to treatment allocation. All biochemical analyses will also be conducted blinded to treatment group.

Plasma glucose will be measured using a glucose oxidase method on the Beckman Auto Analyzer (Beckman, High Wycombe, UK). Serum cholesterol will be analysed using the cholesterol enzymatic assay (Abbott Clinical Chemistry, IL, USA). High density lipoprotein (HDL) cholesterol will be analysed using the ultra HDL assay (Abbott Clinical Chemistry, IL, USA). Serum triglyceride will be analysed using the triglyceride glycerol phosphate oxidase assay (Abbott Clinical Chemistry, IL, USA). Glucose and lipid profile measurements will be undertaken in the same laboratory located within Leicester Royal Infirmary using stable methodology standardised to external quality assurance reference values.

Plasma IL-6 concentration will be measured as described in detail previously (Robinson et al 2009). Briefly, samples diluted 1:5 were analyzed via sandwich enzyme-linked immunosorbent assays (ELISAs) using commercially available anti-human IL-6 antibody pairs (OptEIA, BD Biosciences, Oxford, UK) and an ELISA amplification system (Invitrogen, Paisley UK). This assay will not distinguish between the soluble and receptor-bound IL-6 and therefore measures total IL-6 content. Absorbance will be read at 490 nm (Spectra Max 190, Molecular Devices, USA) and the concentration of the samples determined by relation to a 4-parameter standard curve and adjusted to take account of the dilution factor of the plasma. The intra assay CV of the assay is 5.7% and has an inter assay CV of 7.2%.

Plasma sIL-6R concentration will be measured as described in detail previously (Robinson 2009). Briefly, samples diluted 1:200 will be analysed via sandwich ELISA using anti-human sIL-6R M182 detection antibody and anti-human sIL-6R M5 biotinylated capture antibody (BD Biosciences, Oxford, UK). Absorbance will be read at 450 nm (Spectra Max 190, Molecular Devices, USA) and the concentration of the samples will be determined by relation to a 4-parameter standard curve and adjusted to take account of the dilution factor of the plasma. The intra assay CV is 3.8% and has an inter assay CV of 5.3%.

TNF-α will be measured using a commercially available ELISA kit (RnD systems, Minneapolis, MN, USA) and high sensitivity CRP will be measured on an automated analyser (Pentra 400, Horiba-ABX, Montpellier, France).

All participants will be sent a results letter highlighting their main clinical results after each measurement session. All participants’ results will be sent to their GP.

In addition to the above biochemical variables, we also collect a blood sample for genetic analysis (informed consent will be obtained before these samples are collected). The aim of this sample will be to investigate the associations and interactions of sedentary behaviour, obesity and genes in the development of T2DM. The genetic assessments will be focused on genes for which there are biological plausibility for interaction. The choice of genes and polymorphisms of interest will be decided by an experienced group of researchers. We will genotype all consenting participants for genetic variants in key genes and analyse the data for gene-lifestyle interaction. The demonstration of differential response to lifestyle change by genotype will not only provide greater aetiological understanding, but will also present the opportunity to investigate possibilities to use genotypic data in risk stratification and identification of individuals who have the potential to benefit most from targeted lifestyle modification.

**Anthropometric and demographic data**

- Blood pressure
- Body weight
- Body fat percentage
- Waist and hip circumference
- Height
- Co-morbidities
- Medications.

Arterial blood pressure will be measured in the sitting position (Omron, Healthcare, Henfield, UK); three measurements will be obtained and the average of the last two measurements will be used. Other measures include body weight and body fat percentage (Tanita BC 420SMA, Tanita, West Drayton, UK), waist circumference (midpoint between the lower costal margin and iliac crest), and height to the nearest 0.1 kg, 0.5% and 0.5 cm respectively. Information on current smoking status, medication history, family history and ethnicity will be obtained by self-report.

### Health related quality of life

### EQ-5D

Health-related quality of life will be measured using the EQ-5D (Kind 1998), which is a standardized questionnaire that was developed for use as a measure of health outcomes and defines health in terms of five dimensions: mobility, self-care, usual activities, pain or discomfort, and anxiety or depression. Data from the EQ-5D can be represented either as a health profile (EQ-5D_profile_) or a health index (EQ-5D_utility_) based on time trade-off data from England, which was used to elicit utility weights for the EQ-5D. This instrument can be used to calculate ‘quality adjusted life years’ (QALYs) which are essential to cost-effectiveness analysis.

### Psychological variables and questionnaire data

- Brief Illness Perceptions Questionnaire
- Self-efficacy to increase physical activity and reduce sitting
- Barrier self-efficacy
- Fatigue
- Self-reported measures of sedentary time
- Hospital Anxiety and Depression Score
- Poly cystic ovarian syndrome questionnaire (Pedersen 2007)
- Sleep quality

Several important psychological variables will be measured to establish whether the intervention is targeting the theoretical constructs on which it is based and if so, whether these constructs are determinants of behaviour change as hypothesised.

*Perceptions and perceived knowledge of diabetes risk*

Perceptions and perceived knowledge of diabetes risk will be measured with the validated Brief Illness Perceptions Questionnaire (BIPQ) (Broadbent 2006). This eight item instrument uses an 11 point Likert scale (0 = no effect, 10 = complete effect) to measure five cognitive illness representations (consequences, timeline, personal control, treatment control, and identity), two emotional representations (concern and emotion) and illness comprehensibility (perceived knowledge). The BIPQ provides a practical and comprehensive measurement of determinants identified in Leventhal’s (1980) common sense model, one of the key theoretical models underpinning the content and structure of the education programme. The BIPQ has been shown to have reasonable test-retest reliability and concurrent validity (Broadbent 2006).

*Self-efficacy to increase physical activity and reduce sitting*

Exercise self-efficacy will be measured using the 100% confidence rating scale (from 0% = no confidence to 100% = complete confidence) (Keller 1999). This self-efficacy questionnaire measures participants’ confidence in their ability to undertake any form of moderate- to vigorous-intensity physical activity for 10 minute periods, increasing incrementally from 10 minutes to one hour each day. An overall score is calculated by summing the efficacy scores for each time period and dividing by the number of time periods. Exercise self-efficacy measures using the 100% confidence rating scale have been shown to have good (α > 0.8) internal reliability (McAuley 2003, Cox 2003). To assess self efficacy to reduce sitting time the exercise self-efficacy questionnaire has been modified to focus on confidence to reduce sitting time.

*Barrier self-efficacy*

Participants’ confidence in their ability to self-regulate their exercise behaviour in the face of five commonly identified barriers (tired, bad mood, bad weather, lack of time and holiday) will also be measured (Marcus 1992). This five item questionnaire has been shown to have adequate (α > 0.7) internal reliability (Cox 2003, Marcus 1992). The 100% confidence rating scale will be used; an overall score for self-regulatory efficacy will be calculated by summing the efficacy scores for each barrier divided by the number of barriers.

Fatigue

We will use the Fatigue Scale (Chalder, 1993) to measure fatigue severity. The Fatigue Scale is one of the most widely used measures assessing fatigue and includes 11 items, seven assessing physical fatigue and four assessing mental fatigue. Responses to items are measured using a 4-point Likert-style. A total fatigue score is obtained by adding the score of all 11 items and ranges from 0 (no fatigue) to 33 (maximum fatigue).

*Sedentary time and sleep duration*

We will use a validated questionnaire designed to collect self-report data on time spent in sedentary behaviours (Marshall 2009). We will also devise methods of assessing self-efficacy to reduce sedentary time. Sleep duration has been associated with diabetes risk (Tuomilehto, 2009) and it is feasible that the mediating factor is sedentary behaviour. As such, we will also measure sleep duration and sleep quality via the Pittsburgh Sleep Quality Index (Buysse et al 1989). This is a self-rated questionnaire that assesses sleep quality and disturbances during the previous month.

*Depression and anxiety*

Depression and anxiety will be measured with the 14 item Hospital Anxiety and Depression Scale (Zigmond 2006)

**Dual energy [X-ray](http://www.nhs24.com/content/default.asp?page=s7#x-ray) absorptiometry (DEXA) and** magnetic resonance imaging (MRI) **assessment of body composition**

36 participants (20%) of the STAND study population who are agreeable will be offered imaging to analyse their body fat composition: a whole body dual energy [X-ray](http://www.nhs24.com/content/default.asp?page=s7#x-ray) absorptiometry (DEXA) scan and a single section abdominal magnetic resonance imaging (MRI) scan taken in the L3-L4 region to measure soft tissue body composition, visceral and subcutaneous abdominal fat distribution respectively. As visceral adiposity and overall muscle mass are strongly involved in the aetiology of T2DM these measurements will enable a detailed investigation of the independent effects of sedentary behaviour, physical activity, muscle mass and adiposity in regulating circulating insulin, glucose and adipokines. To our knowledge there are no published data available which investigate the effect of a sedentary behaviour intervention on body composition. Our data may provide preliminary insights into the effect of a reduction of sedentary behaviour on body mass composition. These study measurements will be undertaken in participants who are willing and able to attend University Hospitals of Leicester for these additional tests at each measurement time point. Both scans will be conducted within 14 days of the baseline and follow-up clinical measurement sessions. These scans, by their nature, are unlikely to detect any abnormalities but in the unlikely event that they do our clinical team of doctors will discuss the abnormalities with the participant and their GP, and arrange any further investigations as necessary.

**Remote follow up**

At the end of the study, with their permission, participants will be followed up remotely via office for national statistics tagging and routine general practice data collection.

**SAMPLE SIZE**

The primary outcome of the intervention trial will be reduced sedentary behaviour, measured by an accelerometer (time <100 counts/min as assessed by 1-week accelerometry) at 12 months. The minimum reduction in sedentary behaviour that would yield beneficial metabolic effects has not been determined. Cross-sectional data suggests that each 10% increase in sedentary time is associated with a 3.1cm increase in waist circumference, and that sedentary time is positively associated with clustered metabolic risk (Healy 2008). Using the same dataset, the mean sedentary time is 56.7 hours/week. Therefore, we suggest that a minimum clinically important difference would be 5.67 hours/week, reducing to 51.03 (SD 12.1). Sample size is estimated as 2N = (4(Za+Zb)2s2)/d2 (where d is the true between-arms difference, b is the type II error rate, and a is the type I error rate). Alpha is set at P = 0.05 (Za = 1.96) and power at 80% (b = 0.20, Zb =.842). This results in a required N of 72 in each arm. Incorporating a drop out rate of 25% gives a final N of 89 per arm.

**PROCESS EVALUATION**

Process evaluation will explore the implementation, delivery, context and reach of our STAND intervention. It will also allow us to develop and understanding of its impact on various sub-groups. The questions we wish to address are:

- What is the relation between trial outcome and variation in the extent and quality of the implementation of the intervention?
- What processes mediate the relation between intervention and outcomes?
- Do subgroups (e.g. age, ethnicity etc) differ in their responses to the intervention?

This process will involve quantitative and qualitative methods: questionnaire surveys, focus groups, interviews, researcher observations, and structured field notes during the delivery of the intervention. The identification of key mediating factors will facilitate later understanding and interpretation of the STAND intervention outcomes. Once 1 year follow up data have been collected we will have the opportunity to perform quantitative analysis of the impact of our intervention on various sub-groups (e.g. ethnicity) and mediators (e.g. course educators, location). By integrating our process and outcome data we will be able to develop a fuller understanding of our intervention components (Oakley 2006). This process will allow us to identify the relative merits of the intervention concept/theory and delivery/implementation (Rychetnik 2002).

**DATA ANALYSIS**

The study will be reported according to the CONSORT statement for randomised control trials. Data will be analysed on an intention-to-treat basis (ITT). Data will be analysed using STATA v10 and SPSS v16 software. Descriptive statistics (mean values and frequencies) will be calculated. Histograms will be used to identify any outliers and to test for normality. Robust ANCOVA modelling will be used to look at the difference between groups in change in continuous outcome measures and logistic regression will be used to analyse categorical variables. Results will be disseminated via peer-reviewed publications and presentations at national and international meetings.

#### CONCLUSION

#### This will be the first UK trial to address sedentary behaviour change in a population of young adults at risk of T2DM. The trial will inform behaviour change programmes for this at-risk group, and provide a major new direction of behaviour change alongside the more conventional approach of encouraging increases in moderate-to-vigorous physical activity. The results will provide a platform for the development of a range of future multidisciplinary interventions in this rapidly expanding high-risk population.

**
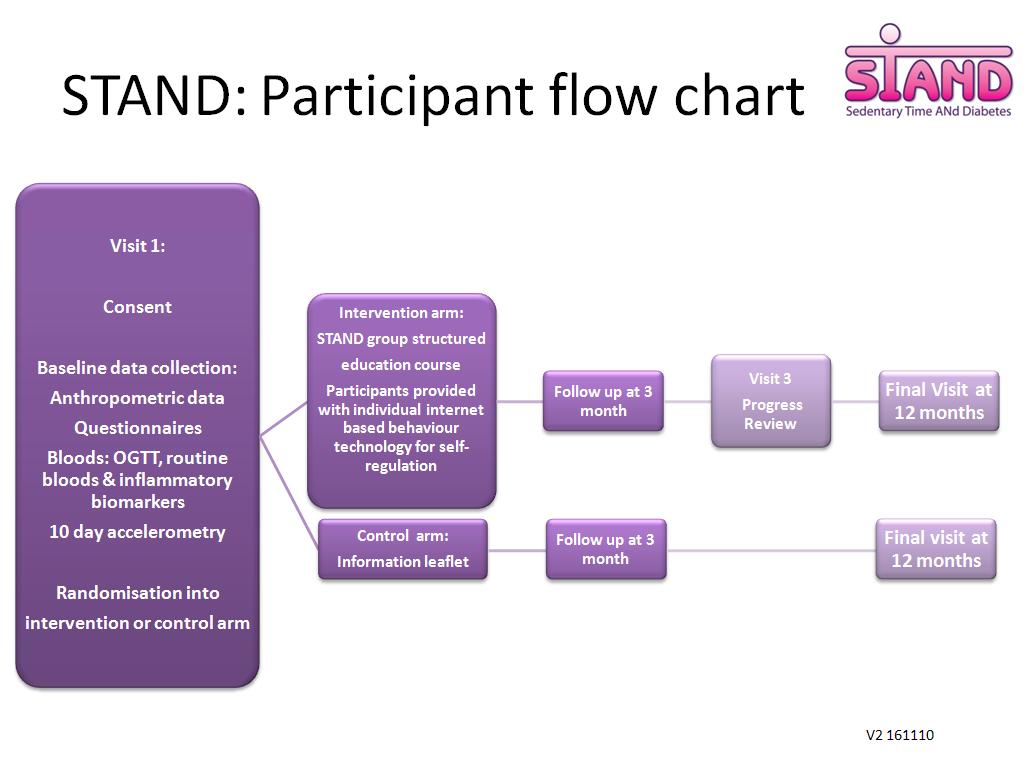
**

**TRIAL STEERING COMMITTEE AND TRIAL SPONSOR**

We will establish a Trial Steering Committee comprising lay members, the project Chief Investigator (Prof Biddle), Principal Investigator (Prof Davies), the project manager, and upto two independent members with academic and clinical trial experience. Membership has yet to be determined. The trial sponsor will be UHL.

**References**

Alberti G, Zimmet P, Shaw J, Bloomgarden Z, Kaufman F, Silink M; Consensus Workshop Group. [Type 2 diabetes in the young: the evolving epidemic: the international diabetes federation consensus workshop.](http://www.ncbi.nlm.nih.gov/pubmed/15220270?ordinalpos=2&itool=EntrezSystem2.PEntrez.Pubmed.Pubmed_ResultsPanel.Pubmed_DefaultReportPanel.Pubmed_RVDocSum)Diabetes Care. 2004 Jul;27(7):1798-811.

Bandura A. Social foundations of thought and action: A social cognitive theory. Englewood Cliffs, NJ: Prentice Hall, 1986.

Bartholomew LK, Parcel GS, Kok G, Gottlieb NH. Intervention mapping: Designing theory and evidence-based health promotion programs. Mountain View, CA: Mayfield, 2001.

Berg AH, Scherer PE. Adipose tissue, inflammation, and cardiovascular disease. Circulation Research 2005;96:939-949.

Bey L, Hamilton MT. Suppression of skeletal muscle lipoprotein lipase activity during physical inactivity: a molecular reason to maintain daily low-intensity activity. Journal of Physiology 2003;551:673-682.

Biddle SJH, Gorely T, Marshall SJ, Cameron N. The prevalence of sedentary behavior and physical activity in leisure time: A study of Scottish adolescents using ecological momentary assessment. Preventive Medicine 2009;48(2):151-155.

Broadbent E, Petrie KJ, Main J, Weinman J. The brief illness perception questionnaire. Journal of psychosomatic research 2006;60:631-637

Buysse DJ, Reynolds CF, Monk TH, Berman SR, Kupfer DJ. The Pittsburgh Sleep Quality Index: a new instrument for psychiatric practice and research. Psychiatry Research 1989:28(2):193-213.

Chaiken S. The heuristic model of persuation. In: Zanna MP, Olson JM, Herman CP, editors. *Social influence: the Ontaio symposium.* 5th ed., NJ: Erlbaum; 1987. p. 3–39.

Chalder T, Berelowitz G, Pawlikowska T, Watts L, Wessely S, Wright D, et al. Development of a fatigue scale. Journal of Psychosomatic Research 1993;37(2):147-153.

Chang P-C, Li T-C, Wu M-T, Liu C-S, Li C-I, Chen C-C, et al. Association between television viewing and the risk of metabolic syndrome in a community-based population. BMC Public Health 2008;8:http://www.biomedcentral.com/1471-2458/8/193.

Cox KL, Gorely TJ, Puddey IB, Burke V, Beilin LJ. Exercise behaviour change in 40 to 65-year-old women: The SWEAT study (sedentary women exercise adherence trial). British journal of health psychology 2003;8:477-95

Craig CL, Marshall AL, Sjöström M, Bauman AE, Booth ML, Ainsworth BE, Pratt M, Ekelund U, Yngve A, Sallis JF, Oja P International physical activity questionnaire: 12-country reliability and validity. Med Sci Sports Exerc 2003;35:1381-1395

Craig P, Dieppe P, Macintyre S, Michie S, Nazareth I, Petticrew M. Developing and evaluating complex interventions: The new Medical Research Council guidance. British Medical Journal 2008;337(sep29_1):a1655-.

Davies MJ, Heller S, Skinner TC, Campbell MJ, Carey ME, Cradock S, et al. Effectiveness of the diabetes education and self management for ongoing and newly diagnosed (DESMOND) programme for people with newly diagnosed type 2 diabetes: Cluster randomised controlled trial. British Medical Journal 2008;336(7642):491-495.

Dunstan DW, Salmon J, Healy GN, Shaw JE, Jolley D, Zimmet PZ, et al. Association of television viewing with fasting and 2-hr post-challenge plasma glucose levels in adults without diagnosed diabetes. Diabetes Care 2007;30:516-522.

Ehtisham S, Barrett TG and Shaw NJ Type 2 diabetes mellitus in UK children – an emerging problem. Diabetic Medicine;2000, 17:867-871.

Ehtisham S, Hattersley AT, Dunger DB, Barrett TG, for the British Society for Paediatric Endocrinology and Diabetes Clinical Trials Group. First UK survey of paediatric type 2 diabetes and MODY. Archives of Disease in Childhood.2004 89:526-529.

Eisenmann JC, Bartee RT, Smith DT, Welk GJ, Fu Q. Combined influence of physical activity and television viewing on the risk of overweight in US youth. International Journal of Obesity 2008;32(4):613-618.

Ekelund U, Brage S, Froberg K, Harro M, Anderssen SA, Sardinha LB, et al. TV viewing and physical activity are independently associated with metabolic risk in children: The European Youth Heart Study. PLoS Medicine 2006;2(12):2449-2456.

Epstein LH, Roemmich JN. Reducing sedentary behaviour: Role in modifying physical activity. Exercise and Sport Sciences Reviews 2001;29(3):103-108.

Febbraio MA, Pedersen BK. Muscle-derived interleukin 6:mechnaisms for activation and possible biological roles. FASEB Journal 2002;16:1335-1347.

Feltbower RG, McKinney PA, Campbell FM, Stephenson CR, Bodansky HJ . Type 2 and other forms of diabetes in 0-30 year olds: a hospital based study in Leeds, UK. Archives of Disease in Childhood 2003; 88: 676-679.

Ford ES, Kohl HW, Mokdad AH, Ajani UA. Sedentary behavior, physical activity, and the metabolic syndrome among U.S. adults. Obesity Research 2005;13:608-614.

Freedson, PS, Melanson E, Sirard J. Calibration of the computer science and applications, inc. accelerometer. Med Sci Sports Exerc 1998;30:777-781

Gorely T, Marshall S, Biddle S, Cameron N. Patterns of sedentary behaviour and physical activity among adolescents in the United Kingdom: Project STIL. Journal of Behavioral Medicine 2007;30(6):521-531.

Hagger MS, Orbell S. A meta-analytic review of the common-sense model of illness representations. Psychology and Health 2003;18:141-184.

Haines L, Wan KC, Lynn R, Barrett TG, Shield JP. Rising incidence of type 2 diabetes in children in the U.K. Diabetes Care. 2007 May;30(5):1097-101. Epub 2007 Jan 26.

Hamilton MT, Hamilton DG, Zderic TW. Role of low energy expenditure and sitting in obesity, metabolic syndrome, Type 2 diabetes, and cardiovascular disease. Diabetes 2007;56:2655-2667.

Hamilton MT, Healy GN, Dunstan DW, Zderic TW, Owen N. Too little exercise and too much sitting: Inactivity physiology and the need for new recommendations on sedentary behavior. Current Cardiovascular Risk Reports 2008;2:292-298.

Healy GN, Dunstan DW, Salmon J, Cerin E, Shaw JE, Zimmet PZ, et al. Breaks in sedentary time: Beneficial associations with metabolic risk. Diabetes Care 2008;31:661-666.

Healy GN, Wijndaele K, Dunstan DW, Shaw JE, Salmon J, Zimmet PZ, et al. Objectively measured sedentary time, physical activity, and metabolic risk: The Australian Diabetes, Obesity and Lifestyle Study (AusDiab). Diabetes Care 2008;31:369-371.

**Hillier TA, Pedula KL.** Complications in Young Adults With Early-Onset Type 2 Diabetes *Diabetes Care* 2003; 26:2999-3005

Hoebe K, Janssen E, Beutler B. The interface between innate and adaptive immunity. Nature Immunology 2004;5:971-974.

Hu FB, Li TY, Colditz GA, Willett WC, Manson JE. Television watching and other sedentary behaviors in relation to risk of obesity and type 2 diabetes mellitus in women. Journal of the American Medical Association 2003;289(14):1785-1791.

Hurling R, Catt M, De Boni M, Fairley BW, Hurst T, Murray P, Richardson A, et al. Using Internet and Mobile Phone Technology to Deliver an Automated Physical Activity Program: Randomized Controlled Study. J Med Internet Res. 2007 Apr–Jun; 9(2): e7.

Jakes RW, Day NE, Khaw KT, Luben R, Oakes S, Welch A, et al. Television viewing and low participation in vigorous recreation are independently associated with obesity and markers of cardiovascular disease risk: EPIC-Norfolk population-based study. European Journal of Clinical Nutrition 2003;57(9):1089-1096.

Jones SA. Directing transition from innate to acquired immunity defining a role for IL-6. Journal of Immunology 2005;175:3463-3468.

Keller C, Fleury J, Gregor-Holt N, Thompson T Predictive ability of social cognitive theory in exercise research: An integrated literature review. The online journal of knowledge synthesis for nursing 1999; 6:2

Khunti K, Stone M, Bankart J, Sinfield P, Talbot D, Farooqi A and Davies M. Physical activity and sedentary behaviours of South Asian and white European children in inner city secondary schools in the UK. *Family Practice* 2007; 24:3:237-244.

Khunti K, Stone MA, Bankart J, Sinfield P, Pancholi A, Walker S, Talbot D, Farooqi A, Davies MJ. Primary prevention of type 2 diabetes and coronary heart disease: action research in secondary schools serving an ethnically diverse UK population. *Journal of Public Health* 2008;30:1:30-7.

Kind, P, Dolan P, Gudex C, Williams A. Variations in population health status: Results from a United Kingdom national questionnaire survey. BMJ 1998;316:736-741

Kirk J, McEvilly A, et al. Prevalence of type 2 diabetes in children in Birmingham. BMJ 2000;322:1428.

Leventhal H, Meyer D, Nerenz D. The common-sense representation of illness danger. In Rachman S (eds) Contributions to Medical Psychology. New York, Pergamon, 1980 pp7-30

Marcus, BH, Selby VC, Niaura RS, Rossi JS (1992) Self-efficacy and the stages of exercise behavior change. Research quarterly for exercise and sport 63:60-66

Marshall AL, Miller Y, Burton N, Brown WJ Measuring Total and Domain-Specific Sitting: A Study of Reliability and Validity Medicine & Science in Sports & Exercise, Publish Ahead of Print. I: 10.1249/MSS.0b013e3181c5ec18

Marshall SJ, Biddle SJH, Gorely T, Cameron N, Murdey I. Relationships between media use, body fatness and physical activity in children and youth: A meta-analysis. International Journal of Obesity 2004;28:1238-1246.

Marshall SJ, Gorely T, Biddle SJH. A descriptive epidemiology of screen-based media use in youth: A review and critique. Journal of Adolescence 2006;29(3):333-349.

McAuley, E, Jerome GJ, Marquez DX, Elavsky S, Blissmer B. Exercise self-efficacy in older adults: Social, affective, and behavioral influences. Annals of Behavioral Medicine 2003;25:1-7

McGavock J, Sellers E, Dean H. Physical activity for the prevention and management of youth-onset type 2 diabetes mellitus: focus on cardiovascular complications. Diab Vasc Dis Res. 2007 Dec;4(4):305-10.

Millett C, Khunti K, Gray J, Saxena S, Netuveli G, Majeed A. Obesity and intermediate clinical outcomes in diabetes: evidence of a differential relationship across ethnic groups. Diabetic Medicine 2008; 25:685-691

O'Connell S, Biddle SJH, Braithwaite R. Are interventions aimed at reducing sedentary behaviours in young people successful? A systematic review. Manuscript in preparation 2009.

Panagiotakos DB, Pitsavos C, Chrysohoou C, Kavouras S, Stefanadis C & ATTICA Study. The associations between leisure-time physical activity and inflammatory and coagulation markers related to cardiovascular disease: the ATTICA Study, *Preventive Medicine 2005;* 40, 432-437.

Parsons TJ, Manor O, Power C. Television viewing and obesity: a prospective study in the 1958 British birth cohort. European Journal of Clinical Nutrition 2008;62(12):1355-1363.

[Pedersen SD](http://www.ncbi.nlm.nih.gov/pubmed?term="Pedersen SD"%5BAuthor%5D&itool=EntrezSystem2.PEntrez.Pubmed.Pubmed_ResultsPanel.Pubmed_RVAbstract), [Brar S](http://www.ncbi.nlm.nih.gov/pubmed?term="Brar S"%5BAuthor%5D&itool=EntrezSystem2.PEntrez.Pubmed.Pubmed_ResultsPanel.Pubmed_RVAbstract), [Faris P](http://www.ncbi.nlm.nih.gov/pubmed?term="Faris P"%5BAuthor%5D&itool=EntrezSystem2.PEntrez.Pubmed.Pubmed_ResultsPanel.Pubmed_RVAbstract), [Corenblum B](http://www.ncbi.nlm.nih.gov/pubmed?term="Corenblum B"%5BAuthor%5D&itool=EntrezSystem2.PEntrez.Pubmed.Pubmed_ResultsPanel.Pubmed_RVAbstract). Polycystic ovary syndrome: validated questionnaire for use in diagnosis. [Can Fam Physician.](javascript:AL_get(this, 'jour', 'Can Fam Physician.');) 2007 Jun;53(6):1042-7, 1041.

Pickup JC, Crook MA. Is type II diabetes mellitus a disease of the innate immune system? Diabetologia 1998;41:1241-1248.

Plasqui, G, Westerterp KR (2007) Physical activity assessment with accelerometers: An evaluation against doubly labeled water. Obesity 15:2371-2379

Rychetnik L, Frommer M, Hawe P, Shiell A. Criteria for evaluating evidence on public health interventions. J Epidemiol Community Health 2002;56:119-27.

Qi L, Rifai N, Hu FB. Interleukin-6 receptor gene, plasma C-reactive protein, and diabetes risk in women. Diabetes 2009;58:275-278.

Sandbaek A, Griffin SJ, Rutten G, Davies M, Stolk R, Khunti K, Borch-Johnsen K, Wareham NJ, Lauritzen T. Stepwise screening for diabetes identifies people with high but modifiable coronary heart disease risk. The ADDITION Study. Diabetologia 2008;51:1127-1134

Stone, E. J., McKenzie, T. L., Welk, G. J., & Booth, M. L. Effects of physical activity interventions in youth: Review and synthesis. *American Journal of Preventive Medicine* 1998;15, 298-315.

Stone M A, Bankart J, Sinfield P, Talbot D, Farooqi A, Davies MJ, Khunti K. Dietary habits of young people attending secondary schools serving a multiethnic, inner-city community in the UK. *Postgrad Medical Journal* 2007;83:115–119

Sugiyama T, Healy GN, Dunstan DW, Salmon J, Owen N. Joint associations of multiple leisuretime sedentary behaviours and physical activity with obesity in Australian adults. International Journal of Behavioral Nutrition and Physical Activity 2008;5:http://www.ijbnpa.org/content/5/1/35.

Tataranni & Ortega. Does adipokine induced activation of the immune system mediate the effect of overnutrition on type 2 diabetes? *Diabetes*, 2005;54, 917-927

Tuomilehto H, Peltonen M, Partinen M, Lavigne G, Eriksson JG, Herder C, Aunola S, Keinänen-Kiukaanniemi S, Ilanne-Parikka P, Uusitupa M, Tuomilehto J, Lindström J, and on behalf of the Finnish Diabetes Prevention Study Group. Sleep Duration, Lifestyle Intervention, and Incidence of Type 2 Diabetes in Impaired Glucose Tolerance: The Finnish Diabetes Prevention Study Diabetes Care 2009 32:1965-1971

Viner RM, Cole TJ. Television viewing in early childhood predicts adult body mass index. Journal of Pediatrics 2005;147:429-435.

Weisberg SP, McCann D, Desai M, Rosenbaum M, Leibel RL, Ferrante AW. Obesity is associated with macrophage accumulation in adipose tissue. Journal of Clinical Investigation 2003;112:1796-1808.

Wijndaele K, Duvigneaud N, Matton L, Duquet W, Delecluse C, Thomis M, et al. Sedentary behaviour, physical activity and a continuous metabolic syndrome risk score in adults. European Journal of Clinical Nutrition 2009;63(3):421-429.

Wittrup HH, Tybjaerg-Hansen A, Nordestgaard BG. Lipoprotein lipase mutations, plasma lipids and lipoproteins, and risk of ischemic heart disease. A meta-analysis. Circulation 1999;99:2901-2907.

Yates T, Khunti K, Bull F, Gorely T, Davies MJ. The role of physical activity in the management of impaired glucose tolerance: A systematic review. Diabetologia 2007;50:1116-1126.

Yates T, Davies M, Brady E, Webb D, Gorely T, Bull F, Talbot D, Sattar N & Kamlesh K (2008). Walking and Inflammatory Markers in Individuals Screened for Type 2 Diabetes, *Preventive Medicine,* In Press, [doi:10.1016/j.ypmed.2008.06.015](http://dx.doi.org/10.1016/j.ypmed.2008.06.015)

Yates T, Davies M, Brady E, Webb D, Gorely T, Bull F, et al. Walking and inflammatory markers in individuals screened for type 2 diabetes. Preventive Medicine 2008;47(4):417- 421.

Yates T, Davies M, Gorely T, Bull F, Khunti K. Rationale, design and baseline data from the PREPARE (Pre-diabetes Risk Education and Physical Activity Recommendation and Encouragement) programme study: A randomized controlled trial. Patient Education and Counselling 2008;doi:10.1016/j.pec.2008.06.010.

Yates T, Khunti K, Bull F, Gorely T, Mandalia, Davies M. Three-month follow-up data from the PREPARE (pre-diabetes risk education and physical activity recommendation and encouragement) programme study (abstract). Diabetes Medicine 2008;25:A48.

Yates T et al. Rationale, design and baseline data from the PREPARE (Prediabetes Risk Education and Physical Activity Recommendation and Encouragement) programme study: A randomized controlled trial. Pat Ed Couns 2008;73:264-271.

Yates T, Khunti K, Bull F, Gorely T, Mandalia P & Davies M. Three-month follow-up data from the PREPARE (Pre-diabetes Risk Education and Physical Activity Recommendation and Encouragement) programme study, *Diabetic Medicine* 2008;25, A48

Yates T, Davies M, Gorely T, Bull F, Khunti K. Effectiveness of a Pragmatic Education Program Designed to Promote Walking Activity in Individuals With Impaired Glucose Tolerance. Diabetes Care 2009 August;32(8):1404-1410.

Zigmond, AS., Snaith, RP. The hospital anxiety and depression scale. Acta Psychiatr Scand 2006; 67:361-70.
