# Supplementary material for: A Randomised Controlled Trial to Reduce Sedentary Time in Young Adults at Risk of Type 2 Diabetes Mellitus: Project STAND (Sedentary Time ANd Diabetes)
Source: PLoS One. 2015 Dec 1;10(12):e0143398. doi: 10.1371/journal.pone.0143398 (PMC4666612; doi:10.1371/journal.pone.0143398)
Supplement: S1 Table — (DOCX) [file pone.0143398.s004.docx]

**Supplementary Table 1**. Characteristics of those who did and did not complete the study, by treatment group

|  | Intervention | | | Control | | |
| --- | --- | --- | --- | --- | --- | --- |
| Variable | Did not complete study (n=30) | Completed study (n=64) | P-value^a^ | Did not complete study (n=25) | Completed study (n=68) | P-value^a^ |
| Age | 32.0 (5.6) | 32.5 (5.4) | 0.68 | 32.4 (5.0) | 33.6 (6.1) | 0.35 |
| Gender (% female) | 23 (76.7) | 43 (67.2) | 0.35 | 20 (80.0) | 42 (61.8) | 0.10 |
| Ethnicity (% black and minority ethnic group) | 4 (13.3) | 14 (21.9) | 0.33 | 3 (12.0) | 16 (23.5) | 0.22 |
| Systolic blood pressure (mmHg) | 116.8 (13.2) | 119.4 (12.7) | 0.36 | 116.7 (14.4) | 123.5 (13.7) | 0.04 |
| Diastolic blood pressure (mmHg) | 81.9 (8.8) | 82.8 (8.6) | 0.63 | 82.4 (9.6) | 85.7 (10.6) | 0.18 |
| BMI (kg/m^2^) | 34.7 (4.7) | 34.6 (5.0) | 0.88 | 34.2 (4.9) | 34.6 (5.1) | 0.73 |
| Obese (%) | 26 (86.7) | 55 (85.9) | 0.92 | 20 (80.0) | 57 (83.8) | 0.67 |
| Waist (cm) | 103.8 (13.7) | 104.0 (14.0) | 0.95 | 100.5 (12.0) | 103.5 (14.6) | 0.36 |
| Body fat (%) | 41.5 (6.8) | 40.6 (7.4) | 0.57 | 41.8 (5.5) | 39.9 (7.5) | 0.25 |
| Fat-free mass (%) | 57.0 (13.5) | 57.3 (14.0) | 0.90 | 54.3 (11.3) | 58.9 (12.6) | 0.11 |
| Cholesterol (mmol/l) | 4.8 (0.9) | 4.9 (0.9) | 0.76 | 5.1 (0.6) | 5.0 (1.1) | 0.76 |
| LDL (mmol/l) | 2.9 (0.7) | 2.9 (0.8) | 0.90 | 3.1 (0.6) | 2.9 (0.8) | 0.31 |
| HDL (mmol/l) | 1.2 (0.3) | 1.2 (0.3) | 0.93 | 1.4 (0.3) | 1.3 (0.3) | 0.28 |
| Triglycerides (mmol/l) | 1.4 (0.6) | 1.5 (0.8) | 0.47 | 1.3 (0.5) | 1.7 (1.7) | 0.21 |
| HbA1c (%) | 5.5 (0.3) | 5.6 (0.4) | 0.18 | 5.5 (0.3) | 5.6 (0.3) | 0.11 |
| Fasting glucose (mmol/l) | 4.8 (0.5) | 4.9 (0.6) | 0.66 | 4.7 (0.4) | 4.8 (0.5) | 0.27 |
| 2-h glucose (mmol/l) | 4.9 (1.5) | 5.6 (1.9) | 0.07 | 5.0 (1.0) | 5.5 (1.5) | 0.10 |
| IMD score | 29.7 (18.6) | 23.4 (16.9) | 0.11 | 27.7 (18.7) | 20.9 (16.1) | 0.09 |
| Current smoker n(%) | 5 (16.7) | 12 (18.8) | 0.81 | 8 (32.0) | 15 (22.1) | 0.33 |
| Unemployed n(%) | 5 (16.7) | 14 (21.9) | 0.56 | 7 (28.0) | 5 (7.4) | 0.01 |

^a^ Tests for differences between study completion/non-completion groups for each treatment arm using t test for continuous variables and chi squared tests for categorical variables
