# Supplementary material for: A Randomised Controlled Trial to Reduce Sedentary Time in Young Adults at Risk of Type 2 Diabetes Mellitus: Project STAND (Sedentary Time ANd Diabetes)
Source: PLoS One. 2015 Dec 1;10(12):e0143398. doi: 10.1371/journal.pone.0143398 (PMC4666612; doi:10.1371/journal.pone.0143398)
Supplement: S2 Table — (DOCX) [file pone.0143398.s005.docx]

**Supplementary Table 2**. Characteristics of those in intervention group who attended/did not attend education session

| Variable | Attended (n=71) | Did not attend (n=23) | P-value^a^ |
| --- | --- | --- | --- |
| Age | 32.4 (5.4) | 32.4 (5.6) | 0.97 |
| Gender (% female) | 49 (69.0) | 17 (73.9) | 0.66 |
| Ethnicity (% black and minority ethnic group) | 12 (16.9) | 6 (26.1) | 0.33 |
| Systolic blood pressure (mmHg) | 119.7 (12.9) | 115.2 (12.2) | 0.14 |
| Diastolic blood pressure (mmHg) | 82.9 (8.5) | 81.2 (9.0) | 0.41 |
| BMI (kg/m^2^) | 34.7 (4.7) | 34.4 (5.6) | 0.80 |
| Obese (%) | 62 (87.3) | 19 (82.6) | 0.57 |
| Waist (cm) | 103.8 (14.0) | 104.4 (13.8) | 0.84 |
| Body fat (%) | 40.9 (6.7) | 40.8 (8.6) | 0.96 |
| Fat-free mass (%) | 57.7 (15.3) | 55.9 (7.5) | 0.60 |
| Cholesterol (mmol/l) | 4.9 (9.3) | 4.8 (0.8) | 0.75 |
| LDL (mmol/l) | 3.0 (0.5) | 2.9 (0.8) | 0.86 |
| HDL (mmol/l) | 1.2 (0.3) | 1.2 (0.3) | 1.00 |
| Triglycerides (mmol/l) | 1.5 (0.8) | 1.3 (0.6) | 0.24 |
| HbA1c (%) | 5.6 (0.4) | 5.5 (0.3) | 0.49 |
| Fasting glucose (mmol/l) | 4.9 (0.6) | 4.7 (0.4) | 0.10 |
| 2-h glucose (mmol/l) | 5.5 (1.8) | 4.9 (1.6) | 0.12 |
| Index of Multiple Deprivation (IMD) score | 26.1 (18.3) | 23.4 (15.5) | 0.54 |
| Current smoker n(%) | 11 (15.5) | 6 (26.1) | 0.25 |
| Unemployed n(%) | 18 (25.4) | 1 (4.4) | ***0.03*** |

^a^ Tests for differences between study completion/non-completion groups for each treatment arm using t test for continuous variables and chi squared tests for categorical variables
