# Supplementary material for: A Randomised Controlled Trial to Reduce Sedentary Time in Young Adults at Risk of Type 2 Diabetes Mellitus: Project STAND (Sedentary Time ANd Diabetes)
Source: PLoS One. 2015 Dec 1;10(12):e0143398. doi: 10.1371/journal.pone.0143398 (PMC4666612; doi:10.1371/journal.pone.0143398)
Supplement: S3 Table — (DOCX) [file pone.0143398.s006.docx]

Supplementary Table 3. Self-reported sedentary behaviours.

|  |
| --- |
| \|  \| Group 1 (Intervention) \| \| Group 2 (Control) \| \|  \|  \| \| --- \| --- \| --- \| --- \| --- \| --- \| --- \| \| Outcome measure \| n \| Mean (95% CI) \| n \| Mean(95% CI) \| Difference (95% CI) \| P-value \| |
| \| Average sitting time per day, hours (IPAQ) \| \| \| \| \| \| \| \| --- \| --- \| --- \| --- \| --- \| --- \| --- \| \| Baseline \| 76 \| 8.53 (6.38, 10.68) \| 76 \| 7.13 (6.44, 7.82) \|  \|  \| \| Change at 3 months \| 55 \| -0.31 (-2.80, 2.17) \| 60 \| 2.45 (-0.41, 5.32) \| -1.10 (-5.09, 2.88) \| 0.581 \| \| Change at 12 months \| 50 \| -3.45 (-6.76, -0.14) \| 53 \| 0.84 (-2.45, 4.14) \| -1.61 (-5.03, 1.82) \| 0.353 \| \| Average sitting time per weekday, hours (Marshall’s sitting questionnaire)* \| \| \| \| \| \| \| \| Baseline \| 85 \| 8.85 (8.19, 9.51) \| 83 \| 9.50 (8.82, 10.19) \|  \|  \| \| Change at 3 months \| 53 \| 0.31 (-0.45, 1.07) \| 56 \| -0.39 (-1.15, 0.36) \| -0.04 (-1.30, 1.23) \| 0.723 \| \| Change at 12 months \| 49 \| 0.35 (-0.55, 1.25) \| 54 \| -0.59 (-1.45, 0.28) \| 0.12 (-1.18, 1.42) \| 0.855 \| \| Average sitting time per weekend day, hours (Marshall’s sitting questionnaire)* \| \| \| \| \| \| \| \| Baseline \| 84 \| 8.72 (8.11, 9.33) \| 81 \| 9.23 (8.61, 9.86) \|  \|  \| \| Change at 3 months \| 52 \| 0.69 (-0.19, 1.56) \| 59 \| -0.30 (-1.22, 0.62) \| 0.14 (-0.94, 1.23) \| 0.793 \| \| Change at 12 months \| 52 \| 0.14 (-0.72, 1.00) \| 55 \| 0.14 (-0.76, 1.03) \| -1.01 (-2.31, 0.29) \| 0.126 \| \| Average sitting time whilst travelling to and from places per weekday, hours \| \| \| \| \| \| \| \| Baseline \| 82 \| 1.07 (0.89, 1.26) \| 76 \| 1.13 (0.94, 1.32) \|  \|  \| \| Change at 3 months \| 51 \| -0.03 (-0.29, 0.23) \| 48 \| -0.01 (-0.30, 0.29) \| -0.20 (-0.57, 0.17) \| 0.277 \| \| Change at 12 months \| 48 \| 0.13 (-0.16, 0.41) \| 49 \| -0.19 (-0.48, 0.10) \| 0.11 (-0.21, 0.44) \| 0.489 \| \| Average sitting time whilst travelling to and from places per weekend day, hours \| \| \| \| \| \| \| \| Baseline \| 78 \| 1.12 (0.96, 1.29) \| 71 \| 1.07 (0.92, 1.23) \|  \|  \| \| Change at 3 months \| 45 \| 0.05 (-0.23, 0.32) \| 48 \| -0.02 (-0.33, 0.30) \| -0.05 (-0.42, 0.31) \| 0.778 \| \| Change at 12 months \| 46 \| 0.17 (-0.18, 0.52) \| 45 \| 0.04 (-0.27, 0.35) \| 0.02 (-0.41, 0.45) \| 0.922 \| \| Average sitting time whilst at work per weekday, hours \| \| \| \| \| \| \| \| Baseline \| 68 \| 3.82 (3.33, 4.30) \| 67 \| 4.27 (3.79, 4.76) \|  \|  \| \| Change at 3 months \| 39 \| -0.33 (-0.83, 0.16) \| 46 \| -0.37 (-0.88, 0.14) \| -0.34 (-1.31, 0.64) \| 0.491 \| \| Change at 12 months \| 41 \| -0.49 (-1.07, 0.08) \| 42 \| -0.32 (-0.89, 0.25) \| -0.42 (-1.45, 0.60) \| 0.411 \| \| Average sitting time whilst at work per weekend day, hours \| \| \| \| \| \| \| \| Baseline \| 42 \| 1.33 (1.10, 1.55) \| 35 \| 1.76 (1.44, 2.09) \|  \|  \| \| Change at 3 months \| 20 \| -0.32 (-1.02, 0.38) \| 23 \| -0.69 (-1.41, 0.04) \| -0.63 (-1.63, 0.37) \| 0.208 \| \| Change at 12 months \| 21 \| 0.14 (-0.59, 0.87) \| 17 \| 0.05 (-0.62, 0.72) \| -1.14 (-2.07, -0.21) \| 0.018 \| \| Average sitting time whilst watching television per weekday, hours \| \| \| \| \| \| \| \| Baseline \| 85 \| 2.61 (2.29, 2.93) \| 81 \| 2.62 (2.37, 2.86) \|  \|  \| \| Change at 3 months \| 52 \| 0.16 (-0.23, 0.56) \| 52 \| 0.15 (-0.59, 0.29) \| 0.18 (-0.48, 0.84) \| 0.583 \| \| Change at 12 months \| 49 \| 0.07 (-0.37, 0.51) \| 51 \| -0.23 (-0.61, 0.14) \| 0.09 (-0.48, 0.67) \| 0.746 \| \| Average sitting time whilst watching television per weekend day, hours \| \| \| \| \| \| \| \| Baseline \| 81 \| 3.60 (3.18, 4.01) \| 78 \| 3.56 (3.19, 3.92) \|  \|  \| \| Change at 3 months \| 47 \| 0.44 (-0.08, 0.96) \| 55 \| 0.15 (-0.37, 0.67) \| 0.14 (-0.52, 0.79) \| 0.678 \| \| Change at 12 months \| 47 \| 0.12 (-0.45, 0.70) \| 50 \| 0.07 (-0.46, 0.61) \| -0.16 (-0.86, 0.54) \| 0.643 \| \| Average sitting time whilst using a computer at home per weekday, hours \| \| \| \| \| \| \| \| Baseline \| 79 \| 1.29 (1.07, 1.51) \| 74 \| 1.53 (1.31, 1.74) \|  \|  \| \| Change at 3 months \| 50 \| 0.09 (-0.26, 0.45) \| 46 \| -0.01 (-0.33, 0.31) \| 0.03 (-0.49, 0.56) \| 0.898 \| \| Change at 12 months \| 44 \| 0.38 (0.001, 0.75) \| 43 \| -0.06 (-0.47, 0.34) \| 0.16 (-0.34, 0.66) \| 0.530 \| \| Average sitting time whilst using a computer at home per weekend day, hours \| \| \| \| \| \| \| \| Baseline \| 78 \| 1.79 (1.50, 2.08) \| 71 \| 1.99 (1.78, 2.21) \|  \|  \| \| Change at 3 months \| 47 \| -0.03 (-0.35, 0.29) \| 48 \| -0.24 (-0.55, 0.07) \| 0.13 (-0.35, 0.61) \| 0.591 \| \| Change at 12 months \| 44 \| 0.04 (-0.40, 0.47) \| 47 \| -0.01 (-0.41, 0.40) \| 0.05 (-0.50, 0.60) \| 0.862 \| \| Average sitting time in leisure time per weekday, hours \| \| \| \| \| \| \| \| Baseline \| 64 \| 1.34 (1.13, 1.54) \| 65 \| 1.33 (1.18, 1.48) \|  \|  \| \| Change at 3 months \| 41 \| 0.18 (-0.25, 0.62) \| 40 \| -0.32 (-0.68, 0.05) \| 0.28 (-0.09, 0.65) \| 0.136 \| \| Change at 12 months \| 32 \| -0.20 (-0.66, 0.27) \| 36 \| -0.17 (-0.54, 0.19) \| -0.05 (-0.55, 0.45) \| 0.846 \| \| Average sitting time in leisure time per weekend day, hours \| \| \| \| \| \| \| \| Baseline \| 71 \| 2.40 (2.14, 2.66) \| 69 \| 2.58 (2.29, 2.86) \|  \|  \| \| Change at 3 months \| 44 \| 0.26 (-0.19, 0.72) \| 46 \| -0.53 (-0.97, -0.08) \| 0.56 (0.03, 1.10) \| 0.038 \| \| Change at 12 months \| 42 \| -0.24 (-0.58, 0.10) \| 41 \| -0.33 (-0.68, 0.02) \| -0.07 (-0.63, 0.49) \| 0.795 \| |

^a^ Adjusted for stratification factors. For accelerometer and ActivPal variables, additionally adjusted for change in wear time.

^b^ Primary outcome.

* NB. If the average hours per weekday across all domains was 0 (n=3 at baseline) or >16 (n=17 at baseline) then all responses to the weekday questions in the Marshall sitting survey were treated as missing. Likewise for the weekend questions.
