# Supplementary material for: A Randomised Controlled Trial to Reduce Sedentary Time in Young Adults at Risk of Type 2 Diabetes Mellitus: Project STAND (Sedentary Time ANd Diabetes)
Source: PLoS One. 2015 Dec 1;10(12):e0143398. doi: 10.1371/journal.pone.0143398 (PMC4666612; doi:10.1371/journal.pone.0143398)
Supplement: S4 Table — (DOCX) [file pone.0143398.s007.docx]

Supplementary Table 4. Secondary outcomes by randomisation group

|  | Group 1 (Intervention) | | Group 2 (Control) | | |  |  | |  |
| --- | --- | --- | --- | --- | --- | --- | --- | --- | --- |
| Outcome measure | n | Mean (95% CI) | n | | Mean(95% CI) | Difference (95% CI) | P-value | |  |
| **Physical activity (accelerometer)** | | | | | | | | |  |
| Average steps per day | | | | | | | | |  |
| Baseline | 75 | 7401.7 (6839.6, 7963.9) | | 80 | 7108.7 (6547.9, 7669.4) |  |  | |  |
| Change at 3 months | 44 | -295.8 (-817.1, 225.4) | | 54 | -16.0 (-422.7, 390.6) | -192.8 (-1264.2, 878.5) | 0.718 | |  |
| Change at 12 months | 37 | 459.2 (-416.7, 1335.2) | | 49 | 66.7 (-656.9, 790.3) | 402.7 (-807.9,1613.4) | 0.507 | |  |
| Average counts per day | | | | | | | | |  |
| Baseline | 75 | 278,228 (259,184, 297,272) | | 80 | 268,462 (246,824, 290,101) |  |  | |  |
| Change at 3 months | 44 | -5747 (-29,548, 18,055) | | 54 | 12,535 (-10,053, 35,122) | -20,950 (-65,650, 23,750) | | 0.350 | |
| Change at 12 months | 37 | 16,104 (-12,496, 44,704) | | 48 | -1151 (-30,819, 28,517) | 18,430 (-32,023, 68,882) | 0.464 | |  |
| Average time in light PA per day, minutes | | | | | | | | |  |
| Baseline | 76 | 154.57 (145.72, 163.43) | | 80 | 155.21 (145.47, 164.94) |  |  | |  |
| Change at 3 months | 45 | -7.49 (-12.4, 3.50) | | 54 | -4.45 (-12.4, 3.50) | -3.38 (-20.8, 14.0) | 0.696 | |  |
| Change at 12 months | 38 | -0.44 (-13.1, 12.3) | | 49 | -9.26 (-19.7, 1.13) | 0.36 (-20.2, 20.9) | 0.972 | |  |
| Average time in MVPA per day, minutes | | | | | | | | |  |
| Baseline | 76 | 58.49 (40.64, 76.34) | | 80 | 46.26 (41.66, 50.87) |  |  | |  |
| Change at 3 months | 45 | -15.8 (-40.3, 8.66) | | 54 | 6.47 (-7.93, 20.9) | -3.93 (-13.2, 5.38) | 0.402 | |  |
| Change at 12 months | 38 | -9.66 (-35.8, 16.5) | | 49 | 5.51 (-9.15, 20.2) | 4.96 (-6.78, 16.7) | 0.397 | |  |
|  |  |  | |  |  |  |  | |  |
| **Physical activity (activPAL)** |  |  | |  |  |  |  | |  |
| Average time spent standing per day, hours | | | | | | | | |  |
| Baseline | 61 | 4.65 (4.35, 4.95) | | 59 | 4.58 (4.36, 4.81) |  |  | |  |
| Change at 3 months | 35 | -0.17 (-0.57, 0.22) | | 36 | 0.12 (-0.28, 0.51) | -0.12 (-0.69, 0.45) | 0.677 | |  |
| Change at 12 months | 34 | -0.42 (-1.02, 0.19) | | 31 | 0.02 (-0.45, 0.48) | -0.30 (-0.97, 0.36) | 0.363 | |  |
|  |  |  | |  |  |  |  | |  |
| **Physical activity (IPAQ)** |  |  | |  |  |  |  | |  |
| Vigorous METs | | | | | | | | |  |
| Baseline | 88 | 1399.0 (1044.2, 1753.8) | | 82 | 1397.2 (986.2, 1808.2) |  |  | |  |
| Change at 3 months | 64 | -191.4 (-591.3, 208.5) | | 59 | -404.5 (-816.6, 7.5) | -2.5 (-536.9, 531.8) | 0.993 | |  |
| Change at 12 months | 54 | -396.9 (-889.4, 95.6) | | 52 | -437.7 (-956.4, 81.0) | -242.0 (-849.9, 365.8) | 0.430 | |  |
| Total METs |  |  | |  |  |  |  | |  |
| Baseline | 75 | 3953.8 (3300.4, 4607.1) | | 72 | 3528.2 (2890.9, 4165.5) |  |  | |  |
| Change at 3 months | 46 | -247.0 (-963.0, 468.9) | | 49 | -582.9 (-1258.0, 92.2) | 185.5 (-830.6, 1201.7) | 0.717 | |  |
| Change at 12 months | 42 | -374.4 (-1300.2, 551.5) | | 43 | -647.5 (-1587.0, 291.9) | 85.7 (-1242.3, 1413.7) | 0.897 | |  |
|  | | | | | | | | |  |
| **Biochemical variables** |  |  | |  |  |  |  | |  |
| Total cholesterol (mmol/l) |  |  | |  |  |  |  | |  |
| Baseline | 94 | 4.85 (4.67, 5.03) | | 92 | 5.00 (4.79, 5.21) |  |  | |  |
| Change at 3 months | 67 | 0.04 (-0.09, 0.17) | | 73 | -0.04 (-0.20, 0.12) | -0.01 (-0.25, 0.24) | 0.967 | |  |
| Change at 12 months | 58 | 0.02 (-0.16, 0.21) | | 64 | -0.20 (-0.38, -0.01) | 0.12 (-0.15, 0.40) | 0.377 | |  |
| LDL cholesterol (mmol/l) |  |  | |  |  |  |  | |  |
| Baseline | 93 | 2.94 (2.79, 3.09) | | 89 | 2.98 (2.83, 3.13) |  |  | |  |
| Change at 3 months | 66 | 0.04 (-0.07, 0.15) | | 67 | -0.07 (-0.18, 0.04) | 0.08 (-0.16, 0.32) | 0.492 | |  |
| Change at 12 months | 57 | 0.05 (-0.09, 0.18) | | 60 | -0.10 (-0.23, -0.02) | 0.13 (-0.10, 0.36) | 0.256 | |  |
| HDL cholesterol (mmol/l) |  |  | |  |  |  |  | |  |
| Baseline | 94 | 1.24 (1.18, 1.30) | | 91 | 1.29 (1.23, 1.36) |  |  | |  |
| Change at 3 months | 67 | -0.02 (-0.07, 0.03) | | 70 | 0.01 (-0.04, 0.06) | -0.05 (-0.13, 0.03) | 0.233 | |  |
| Change at 12 months | 58 | -0.001 (-0.05, 0.05) | | 63 | -0.09 (-0.13, -0.04) | 0.06 (-0.02, 0.14) | 0.122 | |  |
| Triglycerides (mmol/l) |  |  | |  |  |  |  | |  |
| Baseline | 94 | 1.61 (1.31, 1.92) | | 92 | 1.61 (1.31, 1.92) |  |  | |  |
| Change at 3 months | 67 | 0.08 (-0.17, 0.34) | | 73 | 0.08 (-0.17, 0.34) | -0.14 (-0.46, 0.19) | 0.416 | |  |
| Change at 12 months | 58 | -0.04 (-0.24, 0.17) | | 64 | -0.04 (-0.24, 0.17) | -0.17 (-0.42, 0.08) | 0.171 | |  |
| HbA1c (%) |  |  | |  |  |  |  | |  |
| Baseline | 93 | 5.58 (5.51, 5.64) | | 92 | 5.58 (5.51, 5.64) |  |  | |  |
| Change at 3 months | 68 | -0.05 (-0.10, -0.004) | | 73 | -0.05 (-0.10, -0.004) | 0.01 (-0.08, 0.11) | 0.773 | |  |
| Change at 12 months | 58 | 0.06 (0.01, 0.11) | | 63 | 0.06 (0.01, 0.11) | 0.06 (-0.04, 0.16) | 0.207 | |  |
| Fasting glucose (mmol/l) |  |  | |  |  |  |  | |  |
| Baseline | 94 | 4.80 (4.70, 4.90) | | 93 | 4.80 (4.70, 4.90) |  |  | |  |
| Change at 3 months | 69 | 0.17 (0.07, 0.28) | | 76 | 0.17 (0.07, 0.28) | -0.18 (-0.35, -0.01) | *0.040* | |  |
| Change at 12 months | 59 | 0.16 (0.07, 0.26) | | 67 | 0.16 (0.07, 0.26) | -0.01 (-0.20, 0.19) | 0.947 | |  |
| 2-h glucose (mmol/l) |  |  | |  |  |  |  | |  |
| Baseline | 93 | 5.41 (5.13, 5.69) | | 92 | 5.41 (5.13, 5.69) |  |  | |  |
| Change at 3 months | 64 | -0.24 (-0.58, 0.10) | | 72 | -0.24 (-0.58, 0.10) | -0.08 (-0.63, 0.46) | 0.761 | |  |
| Change at 12 months | 51 | 0.25 (-0.34, 0.57) | | 60 | 0.25 (-0.34, 0.57) | 0.10 (-0.65, 0.84) | 0.796 | |  |
| Insulin |  |  | |  |  |  |  | |  |
| Baseline | 91 | 14.11 (11.97, 16.26) | | 90 | 14.11 (11.97, 16.26) |  |  | |  |
| Change at 3 months | 64 | 2.43 (-0.32, 5.18) | | 67 | 2.43 (-0.32, 5.18) | -3.01 (-6.47, 0.45) | 0.088 | |  |
| Change at 12 months | 54 | 1.38 (-2.71, 2.40) | | 61 | 1.38 (-2.71, 2.40) | -2.04 (-5.57, 1.50) | 0.255 | |  |
|  |  |  | |  |  |  |  | |  |
| **Anthropometric variables** |  |  | |  |  |  |  | |  |
| Systolic blood pressure | | | | | | | | |  |
| Baseline | 94 | 118.6 (116.0, 121.2) | | 93 | 121.7 (118.8, 124.6) |  |  | |  |
| Change at 3 months | 70 | -4.3 (-18.3, 9.6) | | 76 | -16.3 (-42.5, 10.0) | 13.3 (-16.7, 43.3) | 0.381 | |  |
| Change at 12 months | 60 | -2.62 (-4.66, -0.59) | | 67 | -3.59 (-5.68, -1.50) | -1.16 (-5.24, 2.92) | 0.572 | |  |
| Diastolic blood pressure |  |  | |  |  |  |  | |  |
| Baseline | 94 | 82.5 (80.7, 84.2) | | 93 | 84.8 (82.7, 86.9) |  |  | |  |
| Change at 3 months | 70 | -0.4 (-14.3, 13.5) | | 76 | -13.9 (-40.1, 12.2) | -15.2 (-14.7, 45.0) | 0.316 | |  |
| Change at 12 months | 60 | -3.28 (-5.20, -1.35) | | 67 | -4.80 (-6.87, -2.72) | -0.49 (-3.66, 2.70) | 0.763 | |  |
| Weight, kg | | | | | | | | |  |
| Baseline | 94 | 98.7 (94.8, 102.5) | | 93 | 98.5 (94.7, 102.2) |  |  | |  |
| Change at 3 months | 70 | 0.6 (-16.9, 18.1) | | 76 | -14.3 (-41.1, 12.5) | 14.3 (-16.1, 44.6) | 0.353 | |  |
| Change at 12 months | 60 | -0.87 (-2.74, 1.00) | | 67 | -1.02 (-2.63, 0.58) | 0.46 (-5.06, 5.97) | 0.869 | |  |
| Body Mass Index kg/m2 |  |  | |  |  |  |  | |  |
| Baseline | 94 | 34.6 (33.6, 35.6) | | 93 | 34.5 (33.5, 35.6) |  |  | |  |
| Change at 3 months | 70 | 2.97 (-10.5, 16.4) | | 76 | -13.2 (-38.1, 11.7) | -13.3 (-15.0, 41.6) | 0.353 | |  |
| Change at 12 months | 60 | -0.21 (-0.83, 0.40) | | 67 | -0.30 (-0.92, 0.31) | 0.41 (-1.19, 2.02) | 0.609 | |  |
| Body fat Percentage |  |  | |  |  |  |  | |  |
| Baseline | 94 | 40.84 (39.38, 42.30) | | 93 | 40.45 (39.01, 41.88) |  |  | |  |
| Change at 3 months | 70 | -14.5 (-43.9, 14.9) | | 76 | -12.1 (-41.0, 16.91) | 0.54 (-38.4, 39.5) | 0.978 | |  |
| Change at 12 months | 60 | -1.63 (-3.04, -0.02) | | 67 | -1.10 (-2.17, -0.04) | -0.25 (-2.72, 2.22) | 0.839 | |  |
| Waist circumference | | | | | | | | |  |
| Baseline | 94 | 103.9 (101.1, 106.7) | | 93 | 102.73 (99.9, 105.6) |  |  | |  |
| Change at 3 months | 70 | -1.56 (-14.5, 11.4) | | 76 | -14.0 (-40.2, 12.2) | 13.4 (-14.7, 41.5) | 0.348 | |  |
| Change at 12 months | 60 | -2.82 (-4.91, -0.73) | | 67 | -2.10 (-4.09, 0.11) | 0.62 (-3.32, 4.55) | 0.755 | |  |
|  |  |  | |  |  |  |  | |  |
| **Health related quality of life and psychological variables** | | | | | | | | |  |
| EQ-5D VAS |  |  | |  |  |  |  | |  |
| Baseline | 88 | 65.12 (61.01, 69.23) | | 82 | 62.01 (58.26, 65.77) |  |  | |  |
| Change at 3 months | 59 | 5.99 (1.65, 10.34) | | 61 | 3.07 (-1.40, 7.55) | -2.59 (-3.29, 8.48) | 0.384 | |  |
| Change at 12 months | 55 | 2.45 (-2.46, 7.36) | | 56 | 4.66 (-0.19, 9.50) | -1.77 (-8.20, 4.67) | 0.585 | |  |
| HADS Anxiety score |  |  | |  |  |  |  | |  |
| Baseline | 94 | 7.15 (6.30, 8.00) | | 93 | 7.92 (7.04, 8.81) |  |  | |  |
| Change at 3 months | 70 | -0.44 (-1.05, 0.17) | | 76 | -0.06 (-0.82, 0.71) | -1.09 (-2.29, 0.10) | 0.072 | |  |
| Change at 12 months | 62 | -0.53 (-1.35, 0.29) | | 67 | -0.24 (-1.11, 0.64) | -0.98 (-2.30, 0.34) | 0.142 | |  |
| HADS Depression score |  |  | |  |  |  |  | |  |
| Baseline | 94 | 4.20 (3.57, 4.84) | | 93 | 5.19 (4.48, 5.91) |  |  | |  |
| Change at 3 months | 70 | -0.11 (-0.75, 0.53) | | 76 | 0.21 (-0.40, 0.83) | -0.74 (-1.80, 0.33) | 0.173 | |  |
| Change at 12 months | 62 | -0.45 (-1.25, 0.34) | | 67 | -0.45 (-1.23, 0.33) | -0.25 (-1.53, 1.02) | 0.691 | |  |
| Confidence in walking 10 mins daily | | | | | | | | |  |
| Baseline | 94 | 9.48 (9.19, 9.77) | | 92 | 9.28 (-8.86, 9.71) |  |  | |  |
| Change at 3 months | 70 | -0.04 (-0.42, 0.34) | | 75 | 0.02 (-0.31, 0.34) | -0.10 (-0.60, 0.41) | 0.70 | |  |
| Change at 12 months | 62 | -1.51 (-4.58, 1.56) | | 66 | 0.01 (-1.92, 1.94) | -2.14 (-5.53, 1.26) | 0.21 | |  |
| Confidence in walking 20 mins daily | | | | | | | | |  |
| Baseline | 94 | 9.05 (8.65, 9.45) | | 93 | 8.87 (8.39, 9.35) |  |  | |  |
| Change at 3 months | 70 | -1.37 (-4.18, 1.44) | | 76 | 0.15 (-1.09, 1.39) | -1.93 (-4.94, 1.08) | 0.21 | |  |
| Change at 12 months | 62 | -0.93 (-4.44, 2.58) | | 67 | -1.65 (-4.82, 1.52) | 0.43 (-4.25, 5.10) | 0.86 | |  |
| Confidence in walking 30 mins daily | | | | | | | | |  |
| Baseline | 93 | 8.55 (8.04, 9.06) | | 90 | 8.37 (7.84, 8.90) |  |  | |  |
| Change at 3 months | 70 | 0.01 (-1.66, 1.68) | | 73 | -1.23 (-3.82, 1.37) | 1.03 (-1.96, 4.02) | 0.50 | |  |
| Change at 12 months | 62 | 0.21 (-2.25, 2.78) | | 65 | -2.81 (-6.54, 0.92) | 3.21 (01.16, 7.57) | 0.15 | |  |
| Confidence in walking 40 mins daily | | | | | | | | |  |
| Baseline | 93 | 7.96 (7.37, 8.55) | | 93 | 7.73 (7.13, 8.34) |  |  | |  |
| Change at 3 months | 70 | -0.27 (-0.85, 0.31) | | 76 | -0.21 (-0.71, 0.28) | -0.13 (-0.98, 0.73) | 0.77 | |  |
| Change at 12 months | 62 | 0.25 (-0.44, 0.93) | | 67 | -0.04 (-0.62, 0.54) | 0.20 (-0.73, 1.13) | 0.67 | |  |
| Confidence in walking 50 mins daily | | | | | | | | |  |
| Baseline | 93 | 7.57 (6.91, 8.22) | | 90 | 7.11 (6.45, 7.76) |  |  | |  |
| Change at 3 months | 70 | -0.48 (-1.19, 0.23) | | 73 | -0.21 (-0.81, 0.38) | -0.20 (-1.14, 0.75) | 0.68 | |  |
| Change at 12 months | 62 | 0.38 (-1.98, 2.72) | | 64 | -1.47 (-4.31, 1.36) | 2.07 (-1.61, 5.76) | 0.27 | |  |
| Confidence in walking 60 mins daily | | | | | | | | |  |
| Baseline | 93 | 7.17 (6.47, 7.87) | | 93 | 6.55 (5.80, 7.30) |  |  | |  |
| Change at 3 months | 70 | -0.70 (-1.50, 0.09) | | 76 | 0.02 (-0.69, 0.73) | -0.47 (-1.50, 0.56) | 0.37 | |  |
| Change at 12 months | 62 | 0.16 (-0.63, 0.95) | | 67 | 0.45 (-0.36, 1.25) | -0.003 (-0.99, 0.98) | 0.996 | |  |
| Confidence in walking 60 mins daily when tired | | | | | | | | |  |
| Baseline | 94 | 6.63 (6.03, 7.22) | | 92 | 6.58 (6.00, 7.15) |  |  | |  |
| Change at 3 months | 70 | 0.02 (-0.69, 0.74) | | 75 | -0.01 (-0.65, 0.63) | -0.23 (-1.06, 0.60) | 0.59 | |  |
| Change at 12 months | 62 | 0.51 (-0.26, 1.29) | | 67 | -0.12 (-0.84, 0.60) | 0.26 (-0.70, 1.22) | 0.60 | |  |
| Confidence in walking 60 mins daily when in a bad mood | | | | | | | | |  |
| Baseline | 94 | 7.84 (7.32, 8.36) | | 92 | 7.63 (7.08, 8.19) |  |  | |  |
| Change at 3 months | 70 | -0.34 (-0.90, 0.23) | | 75 | 0.08 (-0.50, 0.67) | -0.18 (-0.93, 0.57) | 0.64 | |  |
| Change at 12 months | 62 | -0.09 (-0.74, 0.56) | | 67 | 0.001 (-0.59, 0.59) | 0.30 (-0.59, 1.19) | 0.51 | |  |
| Confidence in walking 60 mins daily in bad weather | | | | | | | | |  |
| Baseline | 94 | 6.20 (5.59, 6.82) | | 92 | 5.41 (4.76, 6.05) |  |  | |  |
| Change at 3 months | 70 | -0.71 (-1.35, -0.07) | | 75 | 0.18 (-0.42, 0.77) | -0.32 (-1.13, 0.48) | 0.43 | |  |
| Change at 12 months | 62 | -0.22 (-0.98, 0.55) | | 67 | 0.34 (-0.35, 1.03) | 0.07 (-0.74, 0.89) | 0.86 | |  |
| Confidence in walking 60 mins daily when feel don’t have time | | | | | | | | |  |
| Baseline | 94 | 4.39 (3.77, 5.02) | | 92 | 4.25 (3.65, 4.85) |  |  | |  |
| Change at 3 months | 70 | -0.03 (-0.70, 0.64) | | 75 | 0.38 (-0.25, 1.01) | -0.39 (-1.21, 0.43) | 0.34 | |  |
| Change at 12 months | 62 | -0.04 (-0.79, 0.70) | | 67 | 0.57 (-0.13, 1.28) | -0.34 (-1.29, 0.61) | 0.48 | |  |
| Confidence in walking 60 mins daily when on holiday | | | | | | | | |  |
| Baseline | 94 | 8.18 (7.71, 8.65) | | 92 | 8.08 (7.57, 8.58) |  |  | |  |
| Change at 3 months | 70 | -0.32 (-0.87, 0.24) | | 75 | -0.08 (-0.62, 0.45) | -0.25 (-0.96, 0.46) | 0.48 | |  |
| Change at 12 months | 62 | 0.02 (-1.87, 1.91) | | 67 | 0.02 (-1.87, 1.91) | -1.64 (-5.07, 1.79) | 0.35 | |  |
| Confidence in reducing sitting time by 10 mins daily | | | | | | | | |  |
| Baseline | 92 | 8.79 (8.36, 9.22) | | 90 | 7.77 (7.21, 8.33) |  |  | |  |
| Change at 3 months | 68 | -3.17 (-7.00, 0.66) | | 73 | -0.92 (-4.23, 2.40) | -1.40 (-6.63, 3.84) | 0.60 | |  |
| Change at 12 months | 61 | -5.24 (-11.05, 0.57) | | 64 | -4.08 (-9.42, 1.27) | -0.64 (-8.63, 7.35) | 0.87 | |  |
| Confidence in reducing sitting time by 20 mins daily | | | | | | | | |  |
| Baseline | 92 | 8.46 (8.00, 8.92) | | 92 | 7.48 (6.91, 8.05) |  |  | |  |
| Change at 3 months | 69 | -0.05 (-0.64, 0.53) | | 75 | 0.60 (0.04, 1.17) | 0.17 (-0.54, 0.88) | 0.63 | |  |
| Change at 12 months | 61 | -0.57 (-2.73, 1.59) | | 66 | -1.01 (-4.11, 2.08) | 1.58 (-1.67, 4.83) | 0.34 | |  |
| Confidence in reducing sitting time by 30 mins daily | | | | | | | | |  |
| Baseline | 92 | 7.64 (7.12, 8.16) | | 92 | 6.78 (6.16, 7.40) |  |  | |  |
| Change at 3 months | 69 | -0.30 (-1.00, 0.40) | | 75 | 0.42 (-0.19, 1.03) | -0.10 (-0.98, 0.78) | 0.82 | |  |
| Change at 12 months | 61 | -0.25 (-1.01, 0.5) | | 66 | 0.51 (-0.24, 1.27) | -0.19 (-0.94, 0.56) | 0.62 | |  |
| Confidence in reducing sitting time by 40 mins daily | | | | | | | | |  |
| Baseline | 92 | 6.78 (6.20, 7.35) | | 91 | 6.13 (5.50, 6.76) |  |  | |  |
| Change at 3 months | 69 | -0.01 (-1.64, 1.61) | | 74 | -0.57 (-3.12, 1.98) | 1.00 (-2.10, 4.10) | 0.52 | |  |
| Change at 12 months | 61 | -0.16 (-2.40, 2.08) | | 65 | -0.70 (-3.80, 2.40) | 0.95 (-2.28, 4.19) | 0.56 | |  |
| Confidence in reducing sitting time by 50 mins daily | | | | | | | | |  |
| Baseline | 92 | 6.11 (5.50, 6.73) | | 91 | 5.61 (4.93, 6.29) |  |  | |  |
| Change at 3 months | 69 | -1.64 (-4.93, 1.65) | | 74 | -0.88 (-3.79, 2.03) | -1.22 (-5.63, 3.20) | 0.59 | |  |
| Change at 12 months | 61 | -1.61 (-4.57, 1.36) | | 65 | 0.14 (-1.71, 2.00) | -2.28 (-5.96, 1.39) | 0.22 | |  |
| Confidence in reducing sitting time by 60 mins daily | | | | | | | | |  |
| Baseline | 92 | 5.47 (4.82, 6.13) | | 91 | 5.11 (4.41, 5.82) |  |  | |  |
| Change at 3 months | 69 | -0.05 (-0.72, 0.61) | | 74 | 0.50 (-0.18, 1.19) | -0.20 (-1.20, 0.80) | 0.69 | |  |
| Change at 12 months | 61 | 0.05 (-0.70, 0.80) | | 65 | 0.38 (-0.44, 1.20) | -0.09 (-1.16, 0.97) | 0.86 | |  |
| Confidence in reducing sitting time by 60 mins daily when tired | | | | | | | | |  |
| Baseline | 93 | 4.69 (4.14, 5.24) | | 92 | 4.80 (4.24, 5.36) |  |  | |  |
| Change at 3 months | 69 | -1.47 (-4.98, 2.04) | | 75 | -2.30 (-6.13, 1.53) | 1.72 (-3.45, 6.89) | 0.51 | |  |
| Change at 12 months | 62 | 0.42 (-0.36, 1.20) | | 67 | 1.35 (0.64, 2.05) | -0.78 (-1.60, 0.04) | 0.06 | |  |
| Confidence in reducing sitting time by 60 mins daily when people around you are sitting | | | | | | | | |  |
| Baseline | 94 | 4.12 (3.54, 4.70) | | 91 | 4.33 (3.73, 4.92) |  |  | |  |
| Change at 3 months | 70 | -0.88 (-4.17, 2.41) | | 74 | -1.40 (-4.28, 1.48) | 0.07 (-4.12, 4.27) | 0.97 | |  |
| Change at 12 months | 62 | 1.12 (-1.02, 3.25) | | 66 | -0.50 (-3.36, 2.36) | 1.16 (-2.42, 4.75) | 0.52 | |  |
| Confidence in reducing sitting time by 60 mins daily in bad weather | | | | | | | | |  |
| Baseline | 94 | 5.72 (5.14, 6.30) | | 91 | 5.05 (4.45, 5.65) |  |  | |  |
| Change at 3 months | 70 | 0.23 (-2.04, 2.49) | | 74 | -2.55 (-6.18, 1.07) | 3.44 (-0.70, 7.58) | 0.10 | |  |
| Change at 12 months | 62 | -0.18 (-0.93, 0.56) | | 66 | 0.80 (0.09, 1.51) | -0.58 (-1.55, 0.39) | 0.24 | |  |
| Confidence in reducing sitting time by 60 mins daily when feel don’t have time | | | | | | | | |  |
| Baseline | 93 | 4.96 (4.36, 5.56) | | 91 | 4.86 (4.31, 5.41) |  |  | |  |
| Change at 3 months | 70 | 0.50 (-1.54, 2.55) | | 74 | -2.42 (-6.00, 1.16) | 2.28 (-1.91, 6.48) | 0.28 | |  |
| Change at 12 months | 62 | 0.50 (-1.33, 2.33) | | 66 | -0.87 (-3.56, 1.81) | 0.82 (-2.35, 3.98) | 0.61 | |  |
| Confidence in reducing sitting time by 60 mins daily when on holiday | | | | | | | | |  |
| Baseline | 94 | 7.31 (6.79, 7.82) | | 91 | 7.03 (6.47, 7.59) |  |  | |  |
| Change at 3 months | 70 | -0.19 (-1.97, 1.59) | | 74 | -1.49 (-4.03, 1.05) | 1.67 (-1.22, 4.55) | 0.26 | |  |
| Change at 12 months | 62 | -0.01 (-0.76, 0.74) | | 66 | 0.54 (-0.12, 1.19) | -0.05 (-0.99, 0.89) | 0.91 | |  |

^a^ Adjusted for stratification factors. For accelerometer and activPal variables, additionally adjusted for change in wear time.
