# Supplementary material for: A Randomised Controlled Trial to Reduce Sedentary Time in Young Adults at Risk of Type 2 Diabetes Mellitus: Project STAND (Sedentary Time ANd Diabetes)
Source: PLoS One. 2015 Dec 1;10(12):e0143398. doi: 10.1371/journal.pone.0143398 (PMC4666612; doi:10.1371/journal.pone.0143398)
Supplement: S5 Table — (DOCX) [file pone.0143398.s008.docx]

Supplementary Table 5. Sensitivity analysis

| Outcome measure | Adjusted coefficient at 12 months - per protocol  (95% CI)^a^ | Adjusted coefficient at 12 months – complete case  (95% CI)^a^ |
| --- | --- | --- |
| **Accelerometer measures** |  |  |
| Average sedentary time per day, hours^b^ | -0.21 (-0.72, 0.30) | 0.11 (-0.38, 0.60) |
| Average number of breaks in sedentary behaviour per day | -6.51 (-78.0, 64.0) | -1.20 (-9.93, 7.53) |
|  |  |  |
| **ActivPal measures** |  |  |
| Average sedentary time per day, hours | -0.29 (-1.18, 0.59) | 0.44 (-0.28, 1.17) |
| Average number of breaks in sedentary behaviour per day | 1.52 (-6.20, 9.24) | -1.20 (-9.93, 7.53) |
|  |  |  |

1. Adjusted for stratification factors. For accelerometer and ActivPal variables, additionally adjusted for change in wear time.
2. Primary outcome.
